# Thin-Film Composite Membrane Compaction: Exploring the Interplay among Support Compressive Modulus, Structural Characteristics, and Overall Transport Efficiency

**DOI:** 10.1021/acs.est.4c01639

**Published:** 2024-04-29

**Authors:** Chunyan Xu, Zhongzhen Wang, Yuhang Hu, Yongsheng Chen

**Affiliations:** † School of Resources & Environmental Engineering, Anhui University, Hefei, Anhui 230012, China; ‡ School of Civil & Environmental Engineering, Georgia Institute of Technology, Atlanta, Georgia 30332-0100, United States; § School of Chemical & Biomolecular Engineering, Georgia Institute of Technology, Atlanta, Georgia 30332-0100, United States; ∥ Woodruff School of Mechanical Engineering, Georgia Institute of Technology, Atlanta, Georgia 30332-0100, United States

**Keywords:** thin-film composite membranes, membrane compaction, trade-off, viscoelasticity, Monte Carlo simulation, resistance-in-series

## Abstract

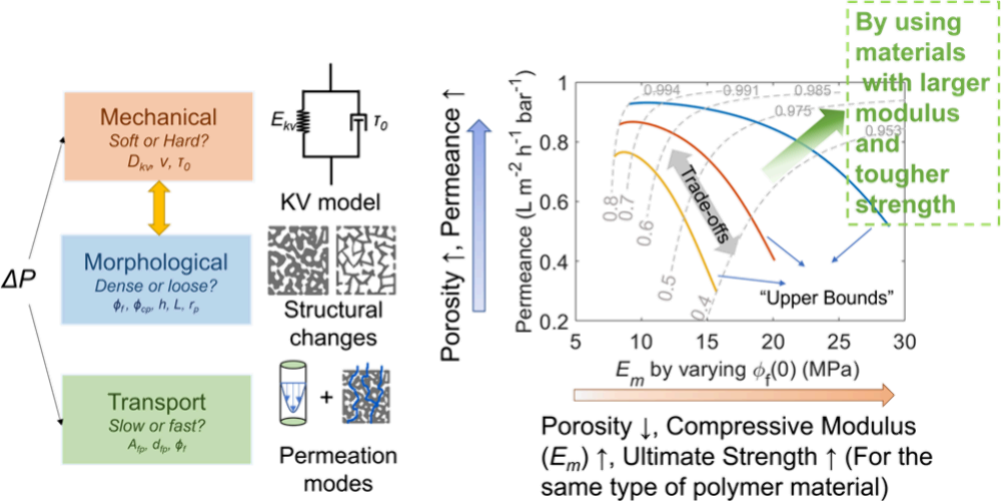

Water scarcity has driven the demand for water production from unconventional sources and the reuse of industrial wastewater. Pressure-driven membranes, notably thin-film composite (TFC) membranes, stand as energy-efficient alternatives to the water scarcity challenge and various wastewater treatments. While pressure drives solvent movement, it concurrently triggers membrane compaction and flux deterioration. This necessitates a profound comprehension of the intricate interplay among compressive modulus, structural properties, and transport efficacy amid the compaction process. In this study, we present an all-encompassing compaction model for TFC membranes, applying authentic structural and mechanical variables, achieved by coupling viscoelasticity with Monte Carlo flux calculations based on the resistance-in-series model. Through validation against experimental data for multiple commercial membranes, we evaluated the influence of diverse physical parameters. We find that support polymers with a higher compressive modulus (lower compliance), supports with higher densities of “finger-like” pores, and “sponge-like” pores with optimum void fractions will be preferred to mitigate compaction. More importantly, we uncover a trade-off correlation between steady-state permeability and the modulus for identical support polymers displaying varying porosities. This model holds the potential as a valuable guide in shaping the design and optimization for further TFC applications and extending its utility to biological scaffolds and hydrogels with thin-film coatings in tissue engineering.

## Abstract 2

A thin-film composite (TFC) membrane support compaction model based on realistic parameters, incorporating viscoelasticity and Monte Carlo flux calculations, illustrates a trade-off relationship between compressive modulus and transport properties.

## Introduction

Freshwater scarcity has been a severe global challenge for decades. Two possible solutions arise from the nature of this problem, involving the generation of freshwater from uncommon natural water resources such as brackish water and seawater, as well as the regeneration and reuse of freshwater from industrial wastewater.^1,2^ Pressure-driven membrane separations, including reverse osmosis (RO) and nanofiltration (NF), are energy-efficient separation technologies to address the increasing demands for water scarcity problems.^1−5^ Seawater desalination, which is a successful example, primarily utilizes membrane-based separations called RO.^6^ These RO membranes operate at relatively high transmembrane pressures to overcome the osmotic pressure difference across the membrane. Compared to thermal-based processes, membrane-based RO processes are much more energy-efficient and have significantly less carbon footprint.^1,6^

Beyond seawater desalination, motivated by the escalating demand for freshwater in multiple industry sectors and the pressing need for energy-efficient wastewater treatment technologies to comply with stringent environmental regulations, membrane-based water treatment processes in dealing with wastewater that have harsher operating conditions than seawater are gaining increasing interests. Various industrial wastewaters have high organic or salt content, such as agricultural wastewater reproduction,^7^ black liquor concentration in the pulp and paper industry,^5,8,9^ and textile wastewater treatment,^10^ can derive substantial environmental benefits by employing pressure-driven membrane separation techniques including RO or NF operated at elevated pressures (10–50 bar). A more challenging scenario is the zero-liquid discharge of brine in the oil and gas industry,^11^ where brine contains significantly higher salt content and is generated in large volumes, which requires even higher operating pressures (50–100 bar).^12−16^ Understanding the impact of transmembrane pressure on membrane structures is essential for achieving high performance and mechanical stability of membranes, particularly under higher operating pressures.

Thin-film composite (TFC) membranes play a dominant role in the above-mentioned RO and NF processes.^3,17−19^ These membranes consist of a thin polyamide skin layer (<200 nm), which provides solutes/solvents selectivity, and a thick macroporous polymer membrane (∼200 μm) that serves as mechanical support to withstand the transmembrane pressure (5–70 bar) while enabling fast solvent transport.^8,20^ However, a major disadvantage is that though pressure provides a driving force or induces a chemical potential for transport,^21^ it also results in TFC membrane compaction as the macroporous support polymer loses its porosity with time and, consequently, flux declines.^5,9,15^ The flux decline caused by compaction is inherent and necessitates higher operating pressure to maintain desired flux levels, leading to increased energy consumption.^5^

The investigations on membrane compaction have been ongoing for several decades. Initial studies indicate that compaction may occur in both the selective and the support layers. Given the macroporous nature of the support layer, compaction will preferentially happen in support membranes as they undergo greater stress distributions, as evidenced by multiple studies.^22−25^ Based on these results, Pendergast et al. incorporated various nanoparticles into the polysulfone support layer of the TFC membrane.^26^ The resultant modified membranes demonstrated reduced compaction and enhanced steady-state permeate flux due to the better maintenance of the support macrovoid upon the addition of nanoparticles. Davenport et al. used positron annihilation lifetime spectroscopy to investigate the pore sizes of the active layer and multiple characterization techniques to understand commercial TFC membrane compaction, particularly under higher operational pressures.^15^ The results have shown that compaction mainly occurs in the support layer, while the polyamide layers remain unchanged, and the adjustment and optimization of the support membrane porosity, pore structure, and hydrophilicity are proposed as solutions to membrane compaction. Molecular dynamics simulations have also shown that the active layer only faced very slight compaction at a pressure as high as 600 bar, which only slightly affects the flux.^27^ Recently, Zhao et al. investigated TFC membranes with rough and smooth polyamide (PA) morphologies and support with dense and loose structures. For denser support structures with rough PA surfaces, compaction happens in both the PA layer and support substrates, while compaction does not occur in PA surfaces with smoother structures but only in the support substrates. Loose supports face even stronger compactions.^28^ Aghajani et al. precisely imposed compressive strains on TFC membranes by nanoimprint lithography, characterized the membranes by SEM and AFM, and quantified the resistance of the supports by modeling. They found that the active layer is not changing explicitly, but the support membrane exhibited significant compressive strains and transport resistance increments.^29^ In another work, they further proved that the support membrane could contribute to as high as 45% of the total transport resistance in TFC when pressure is around 40–80 bar.^30^ All of the works have highlighted the pressure-induced deformation of the support membrane and the relationships between the shrinking support pores and the flux decline. It is suggested that for the same kind of support polymer material, denser structures (smaller porosity) are advantageous and preferred to mitigate compaction.

Addressing support compaction by adjusting the porosities of the macroporous support polymers appears to be a viable solution, but it raises additional concerns. On one hand, reducing the initial porosity during manufacturing (increasing polymer density) can enhance the compressive modulus, mitigating compaction. However, reducing porosity may lead to a reduction in initial flux if the macroporous structures, such as pore interconnectivity and types of pores, remain unchanged during manufacturing. On the other hand, increasing initial porosity improves initial permeance but exacerbates compaction and may potentially deteriorate the mechanical strength of the membrane.^15^ It is evident that a trade-off relationship exists between some of the mechanical properties (such as compressive modulus) and transport properties for separation membranes, and the improvement of transport properties through increased porosity inevitably leads to a compromise in compressive modulus and vice versa.^14^ The same issue also limits the application of a broad range of functional materials involving macroporous polymers, such as biological scaffolds in tissue engineering,^31−33^ hydrogels,^34−36^ polymer foam armors,^37^ and heat-transfer porous materials.^38^ In all of these applications, it is crucial for the porous polymer to possess both mechanical robustness and sufficient porosity to facilitate fast transport or permeation,^34,35,39−41^ but this trade-off phenomenon has not been fully elucidated.

Hence, simulating the complex interplay between compressive modulus, membrane structure, and transport properties of TFC membranes could provide mechanistic insights into the membrane compaction mechanism and give guidance toward future TFC design and many other above-mentioned applications. Previous compaction models have relied on empirical parameters to fit the flux vs time data^42−44^ but have not incorporated realistic physical parameters of the support polymer, such as porosity, pore size, and compliance. Consequently, these models provide limited insight into the evolution of support morphologies during compaction.

This study presents the development of a comprehensive membrane compaction model that establishes a correlation between real-time permeance and physically realistic mechanical and structural parameters. To enhance the visualization of compaction dynamics, colormap animations are employed, providing insights into the distribution of permeance variations during the compaction process. By incorporating these realistic parameters, our compaction model contributes to a deeper understanding of the intricate relationships among the compressive modulus, structural characteristics, and transport behavior in macroporous materials. The insights gained from the compaction model not only can guide future optimizations aimed at enhancing the performance of TFC membranes^16,45,46^ but also inspire the design of scaffolds and hydrogels with similar structures to maximize their effectiveness.

## Methods

**Figure 1:**
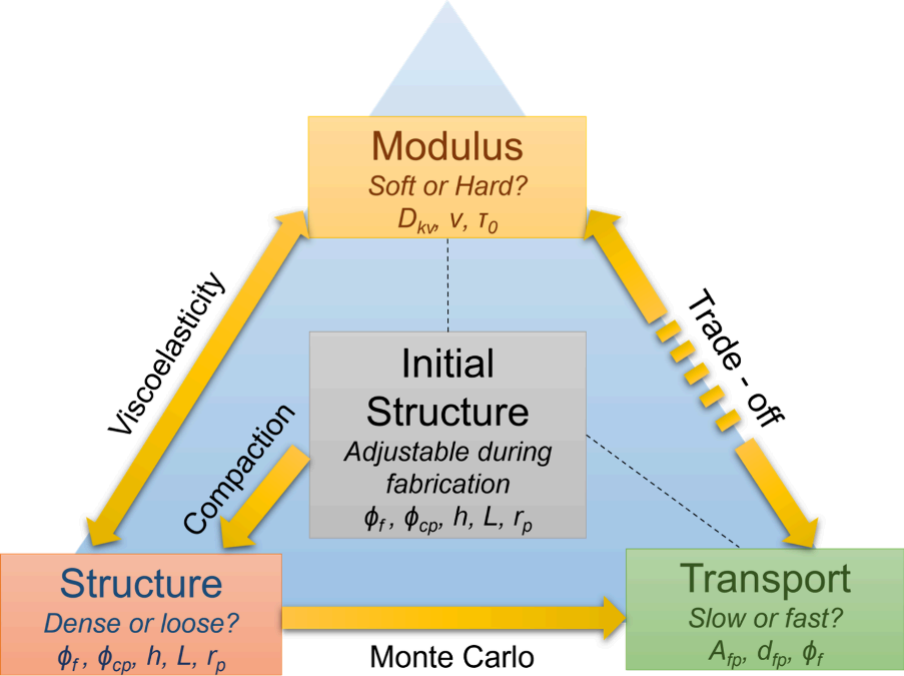
Triangular relationship between steady-state compressive modulus, structural characteristics, and transport efficiency of the TFC membranes during membrane compaction, along with their connections to the initial precompaction membrane support structures when *t* = 0. Here, *D*_kv_ is the creep compliance of the Kelvin–Voigt element of the bulk polymer, ν is the Poisson’s ratio of the foam polymer, τ_0_ is the response time, ϕ_f_ is the porosity of the foam polymer, ϕ_cp_ is the cylindrical pore volume fraction, *h* is the active layer thickness, *L* is the support thickness, and *r*_p_ is the pore size of the cylindrical pores.

**Figure 2:**
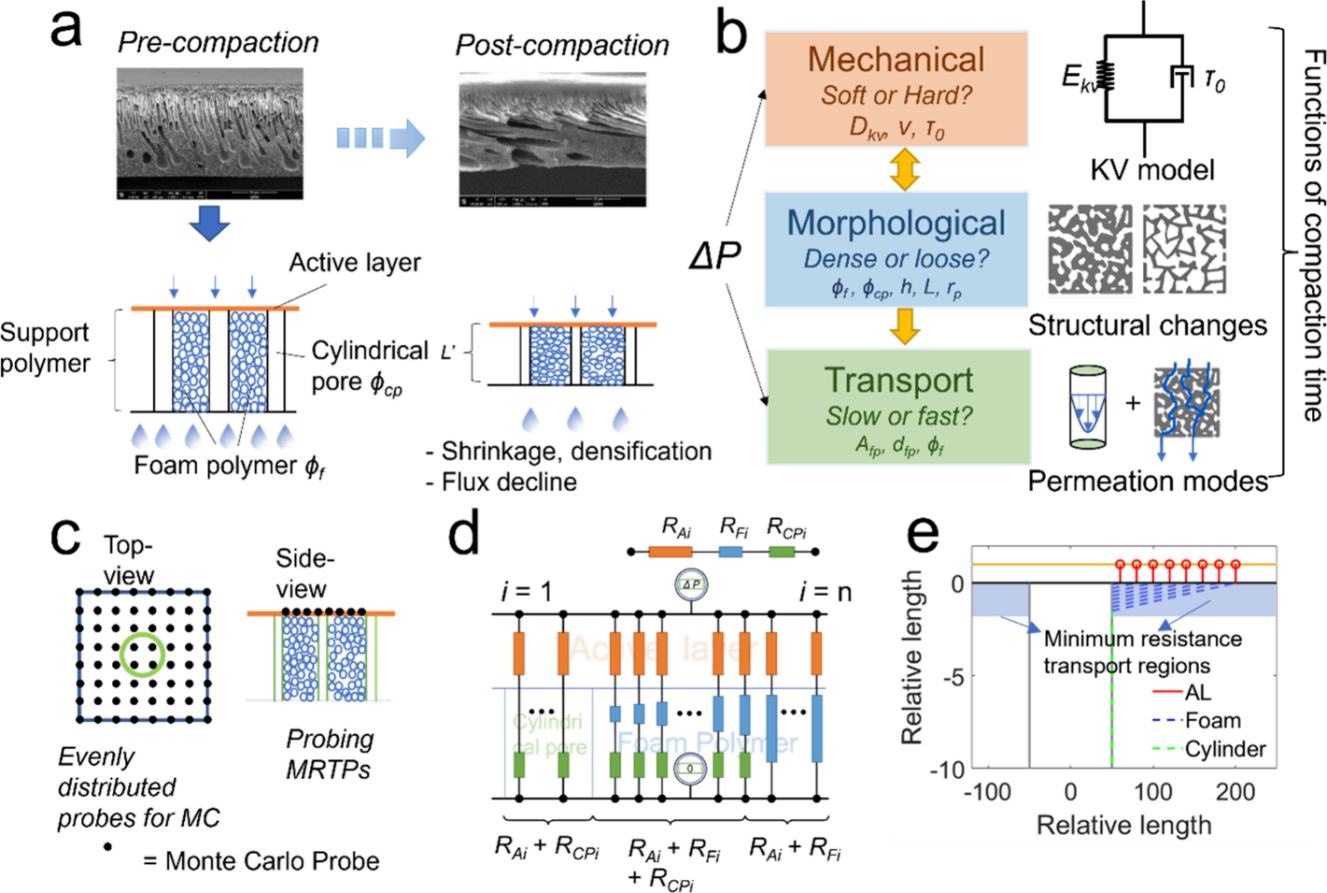
Thin-film composite (TFC) separation membrane structures and modeling techniques. (a) shows the structures of TFC membranes before and after compaction as well as simplified model structures used for analysis. SEM images are polysulfone membranes before and after 10 bar compaction. Reproduced from ref (47) used under the terms of a Creative Commons license (CC BY-NC 4.0 Deed). (b) Models developed through the interplay between mechanical properties, structural characteristics, and transport efficiency of the membranes. (c) The entire resistance of the TFC membrane is evaluated using a uniformly square-mesh sampled Monte Carlo integration approach on a periodic simulation cell (28 μm × 28 μm). Each water probe in the Monte Carlo simulation follows the minimum resistance transport pathway (MRTP) through the membrane. For each periodic cell, we used 10,000 MC probes to ensure the accuracy of the calculation. (d) Evaluation of the transport resistance for probes at different locations within the membrane. (e) Distributions of the MRTPs in the support membrane. It seems that for the MC probes distributed atop the foam polymer their MRTPs pass through a thin layer in the foam polymer located just below the active layer and at the very top of the foam polymer support, constituting a transport preference zone. We refer to this zone as the “interface layer” in this work due to its thin thickness.

The interplay among modulus, structural, and transport properties of TFC membranes during compaction can be visualized as a triangular relationship in Figure 1. The initial structure of TFC was determined by the manufacturing conditions and served as the starting point of analysis. To simplify the initial TFC structure before compaction, we focused on the support membrane as previously discussed.^15^ Therefore, we assume that the active layer thickness (*h*) and permeability (*P*_a_) are fixed, while the structural parameters of the support membrane vary with time. Figure 2a left shows the initial precompaction cross-sectional structures of the TFC membranes.^47^ From the cross-sectional SEM images, it is observed that the support membrane consists of both “sponge-like” pores and “finger-like” pores, which originated from the phase separation kinetics.^48^ To simplify the modeling, we represented the entire support membrane as a flat sheet with uniform thickness (*L*). The “finger-like” pores are approximated as cylindrical pores (with a fraction represented as ϕ_cp_) that traverse the entire thickness of the support membrane perpendicular to the membrane surface. The “sponge-like” pores are represented as open-cellular foams with identical sizes, constituting the rest of the support membrane with porosity ϕ_f_. This simplification defines the initial conditions of compaction when *t* = 0 is at the center of the “compaction triangle” in Figure 1.

As compaction progresses with time, the polymer foam undergoes deformation due to transmembrane pressure, resulting in a decrease in membrane thickness and lateral expansion into the cylindrical pores at a Poisson’s ratio (ν). This leads to the reduction in membrane thickness (*L*′), densification of the foam polymer, and shrinkage of the cylindrical pores (Figure 2a right). Thus, the first challenge of the modeling is to establish real-time relationships between structural parameters ϕ_f_(*t*), ϕ_cp_(*t*), and compressive strain ε(*t*) during compaction (linkage arrow “Compaction” in Figure 1). By applying mass conservation and considering geometric relationships^37^ and neglecting any forces in the membrane plane, the relationship between real-time polymer foam density (ϕ_f_(*t*)) and the strain of the polymer (ε(*t*)) is presented in eq 1.
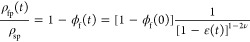
1where ρ_fp_(*t*) is the real-time density of the foam polymer, ρ_sp_ is the density of the solid polymer, and ϕ_cp_(*t*) is the cylindrical pore volume fraction.

The second task is to connect the real-time strain ε(*t*) of the support membrane with the mechanical property of the TFC membrane (linkage arrow “Viscoelasticity” in Figure 1). The viscoelastic behavior of the membrane is modeled as a Kelvin–Voigt element.^49^ The real-time strain ε(*t*) is related to the real-time membrane creep compliance (*D*_m_(*t*)), which depends on the overall polymer density (a function of ϕ_f_(*t*) and ϕ_cp_(*t*)) and the creep compliance (*D*_sp_) of the nonporous solid polymer (eq 2)
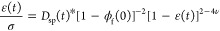
2where σ is the normal stress on the membrane.

Once the real-time support structural parameters ϕ_f_(*t*), ϕ_cp_(*t*), and ε(*t*) are solved, the last challenge is to evaluate the transport resistance of the real-time support structures, and subsequently, the membrane permeate flux (linkage arrow “Monte Carlo” in Figure 1). To analyze the real-time transport resistance and the flux/permeance of the membrane, we used an analytical, nonweighted Monte Carlo (MC) integration approach,^50^ combined with resistance-in-series model,^51,52^ which are presented in Figure 2c,d as well as eqs 3–5.
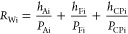
3
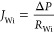
4
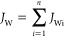
5where *R*_wi_ is the resistance of the water probe *i*, *R*_wi_ is the total resistance, and *J*_wi_ is the local water flux of the water probe *i*, *h*_Ai_, *h*_Fi_, and *h*_CPi_ are minimum resistance transport lengths in the active layer, foam polymer, and the cylindrical polymer, respectively. *P*_Ai_, *P*_Fi_, and *P*_CPi_ are solvent permeabilities in the active layer, foam polymer, and cylindrical polymer, and their units are in L m^–1^ h^–1^ bar^–1^, respectively. To evaluate *J*_wi_, the simulation area of the membrane was divided into periodic square cells (28 μm × 28 μm for the baseline values in Table S1), with each cell containing a cylindrical pore at its center (Figure 2c). The MC probes were evenly distributed throughout the membrane and assumed to follow the minimum resistance transport pathway (MRTP) as they traversed the membrane.^50^ This means that the MC probes choose their pathways by selecting the one with the least transport resistance from the top surface of the active layer to the very bottom of the support layer. Thus, the MRTPs probes initially crossed the active layer and then entered either the cylindrical pores or the foam polymer or a combination of both depending on their location (Figure 2d). The algorithm for calculating the MRTPs is described in the Supporting Information. It involves solving a mathematical moving points optimization problem: finding the minimum of the objective function (*R*_wi_ in eq 3) by optimizing the transport lengths (*h*_Ai_, *h*_Fi_, and *h*_CPi_) in each part of the TFC membrane (Figure S3).

The flux can then be calculated by the average of each individual MC probe in the periodic simulation cell with one cylindrical pore at the center. By employing the MC approach in eqs 3–5, the *J*_w_ vs *t* correlation can now be derived by using all these realistic structural parameters, which completes the compaction modeling. Details regarding the derivations of eqs 1–5 are available in the Supporting Information. This comprehensive model allows us to analyze the dynamic relationships among mechanical, structural, and transport properties during TFC membrane compaction, which completes the entire “compaction triangle” in Figure 1.

When evaluating the distributions of MRTPs, we find that all of the MC probes located above the foam polymer preferred to transport through a very narrow part at the very top of the foam polymer (with a thickness of less than 2 h, approximately 400 nm in this model), as presented in Figure 2e. The distributions of MRTPs result from the relative permeabilities of the different parts of polymers. The cylindrical pores had sizes in the range of several micrometers, while the foams consisted of cells ranging from 10 to 1000 nm in size.^16^ Additionally, the active layer could be nanoporous or nonporous.^3^ Considering the relative pore sizes, the permeability sequence of the cylindrical pore, foam polymer, and active layer should be *P*_cp_ ≫ *P*_f_ ≫ *P*_a_ (Table S3). It is this permeability sequence that influences the distributions of MRTPs throughout the membrane. Hence, we hypothesized that the transport resistance increases in this thin surface layer of the support membrane contributed majorly to flux decline during compaction and will be termed the “interface layer” in this work. Note that according to our model assumptions, this “interface layer” is part of the foam polymer and shares the same porosity and pore size as the foam polymer.

## Results and Discussions

**Figure 3:**
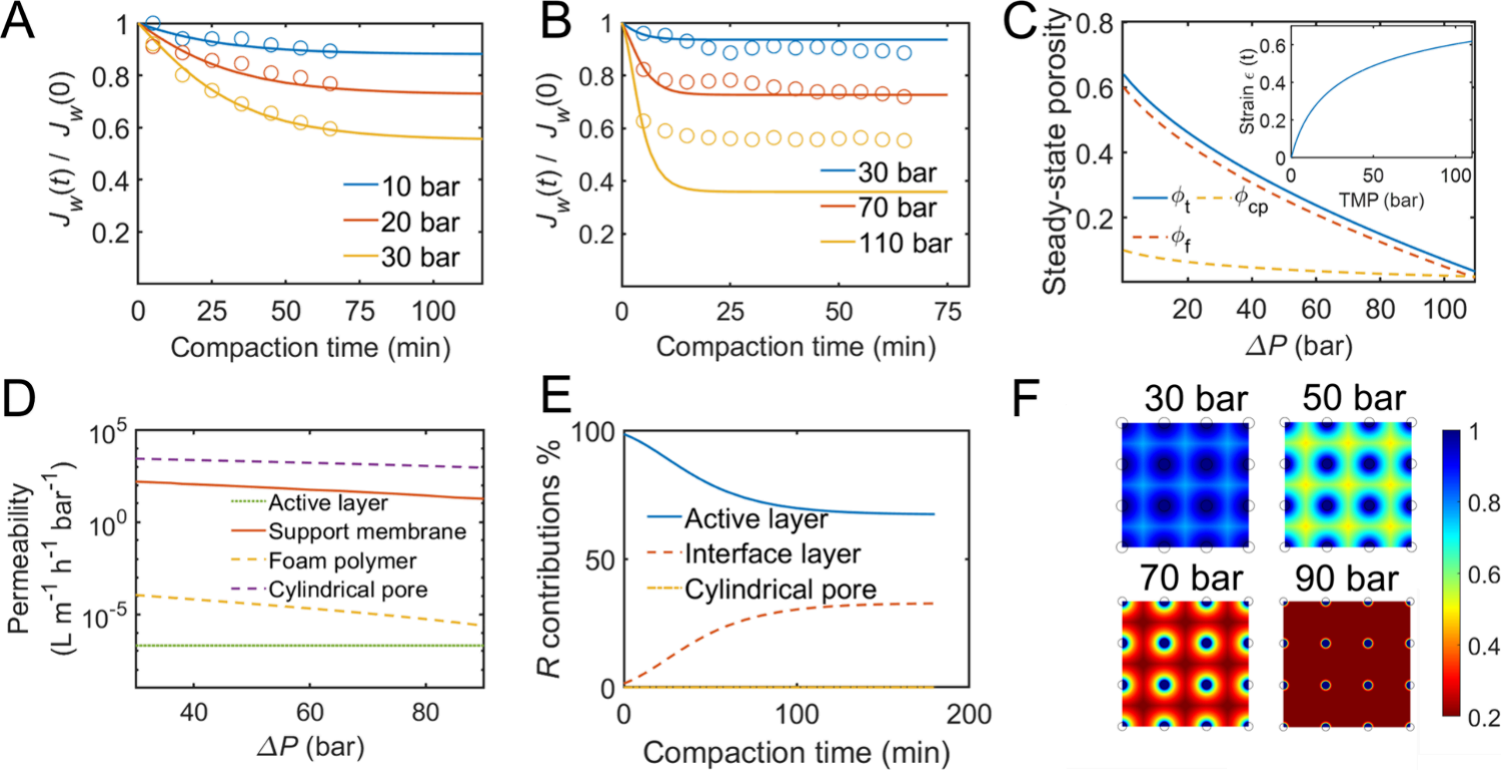
Effect of transmembrane pressure (Δ*P*) on TFC membrane compaction. (A) The fitting of the compaction flux vs time data for the NF-90 membrane by Hussian et al. (ref (44) using *D*_kv_ = 6.4 × 10^–8^ Pa^–1^, τ_0_ = 1500 s). (B) The fitting of the compaction flux vs time data for the SW-30 membrane by Davenport et al. (ref (15) using *D*_kv_ = 3.3 × 10^–8^ Pa^–1^, τ_0_ = 200 s). In parts (A) and (B), the initial permeance of the TFC membrane at *t* = 0 is set to unity, and the normalized permeances data points are fitted using our model. (C) The steady-state porosities ϕ_t_(*t*_s_), ϕ_cp_(*t*_s_), and ϕ_f_(*t*_s_), as functions of Δ*P*. The inset graph: the steady-state strain of the support membrane as a function of Δ*P*. (D) The steady-state permeabilities of the foam polymer (*P*_f_), cylindrical pore (*P*_cp_), and the whole support membrane (*P*_t_), as functions of Δ*P*. The permeability of the active layer is assumed to be independent of the Δ*P*. (E) The resistance contributions percentage of the active layer, the “intermediate layer”, and the cylindrical pores during the TFC compaction process. (F) Top-view colormaps of the steady-state permeance distributions at different Δ*P* levels ranging from 30 to 90 bar. The black solid circles represent the cylindrical pores at the center of the periodic modeling cell.

We first conducted a validation of our model using experimental compaction data obtained from NF-90 and SW-30 membranes, as reported in the literature^15,44^ (Figure 3A,B). By adjusting two parameters for each membrane type, namely, the polymer creep compliance *D*_kv_ and the initial response time (τ_kv,0_), our model successfully matched with the experimental data up to a pressure of 70 bar. The specific values of the fitting parameters for each membrane type can be found in the caption of Figure 3, while the remaining mechanical and structural parameters used in the model are provided in Table S1.^37,49,53,54^ By accurately capturing the compaction behavior of NF-90 and SW-30 membranes, our model can be confidently applied to investigate and predict the compaction dynamics of similar membranes in various operating conditions. In addition, we have also fitted EPS2 (a brackish water membrane) and SWC4 membranes^28^ in Figure S5, as well as NF-270 membrane with sponge-like pores support^55^ (Figure S6c), which further validates the applicability of this model.

Utilizing the fitting results of our compaction model, we now focus on predictive simulations to investigate the individual contributions of various mechanical and structural factors during compaction. We set *D*_kv_ = 5.0 × 10^–8^ Pa^–1^, τ_kv_ = 15 min, and employ the baseline values for the remaining structural parameters and permeability coefficients from Table S1. In Figure 3C, we plot the steady-state total porosity, ϕ_t_(*t*_s_), foam porosity ϕ_f_(*t*_s_), and the cylindrical pore fraction ϕ_cp_(*t*_s_) as functions of the transmembrane pressure. The decrease in ϕ_f_ is a consequence of compression experienced by the foam polymer cells. Simultaneously, the support membrane will undergo lateral expansion perpendicular to the applied stress, following the established three-dimensional stress–strain law. The O-ring suppresses the membranes and maintains its leak-tight condition, which prevents the membrane from expanding laterally beyond the O-ring boundary. Therefore, the foam polymer has to expand into the cylindrical pores, causing their shrinkage and resulting in a reduction in ϕ_cp_.^56^ The inset of Figure 3C depicts that the steady-state compressive strain increases with pressure, indicating more severe compaction at higher pressures. We then calculated and plotted the steady-state permeabilities of the foam polymer (*P*_f_), cylindrical pores (*P*_cp_), and entire support polymer (*P*_t_) against the transmembrane pressure in Figure 3D, with *P*_a_ included as a reference. As depicted, *P*_cp_, *P*_f_, and *P*_t_ decrease as the pressure increases, aligning with the trends observed in Figure 3C. Even under pressure up to 90 bar, the steady-state permeabilities still follow the sequence *P*_cp_ ≫ *P*_f_ ≫ *P*_a_.

Using the obtained real-time permeabilities, we further examined the transport resistances contributed by different parts of the membrane. Figure 3E shows that at 50 bar pressure, the resistance contributed by the “interface layer” increased from ∼1 to 30% during compaction, which verified our previous hypothesis (Figure 2e). This result highlights the influential role played by the modulus and structural properties of the “interface layer” during compaction. To mitigate the flux decline associated with compaction, modifications to this “interface layer”, such as the addition of an “intermediate layer” or “gutter layer” composed of mechanically porous materials, could be implemented. While existing research on incorporating gutter layers in TFC membranes has reported increased steady-state permeate flux,^57,58^ further examination is necessary to determine if this flux increase is indeed a result of reduced membrane compaction. Figure 3F shows the colormap of steady-state permeance distribution in the membrane at different pressures. From 30 to 90 bar, the local permeance for water probes atop the cylindrical pores undergoes minimal changes, as indicated by the dark blue color within and around the circle. However, the local permeances atop the foam polymer significantly decrease, transitioning from blue to yellow and eventually to red. This further underscores the significance of the “interface layer” in the membrane flux decline during compaction.

In addition to examining the cylinder and foam (C + F) support structures, we have also investigated the effect of transmembrane pressure on the compaction of foam-only (F-only) structures, representing support membranes with only sponge-like pores, as depicted in Figure S6. We used the same set of parameters as those tabulated for the C + F structures in Table S1. Assuming υ = 0 (since the membrane cannot be expanded into the pores anymore), we have also modeled membranes with F-only structures. Figure S5 presents these results, comparing the foam polymer supports with the cylinder and foam (C + F) supports and validating the data with NF-270 membranes. Figure S6a compares the C + F structures with the F-only structures, revealing that the F-only structures exhibited smaller initial flux and a more significant flux decline due to the absence of cylindrical pores, which facilitate mass transport. In Figure S6b, it is shown that F-only structures have similar strains as the C + F membrane under the same Δ*P*. This similarity arises because the foam component possesses the same properties in the simulation, resulting in similar levels of compaction between the two structures. Despite the similar levels of compaction, the flux decline for the F-only structures is much greater, further emphasizing the importance of finger-like pores in the support membranes.

The creep compliance of the solid polymer (*D*_kv_) is shown to have a substantial impact on compaction (Figure S7a). Stiffer polymers with larger *D*_kv_ values experience less compaction, while more compliant polymers exhibit the opposite behavior. This result implies the importance of using stronger polymer materials or the reinforcement of the polymer supports through the addition of nanoparticles.^56^ The Poisson’s ratio (υ) was found to have minimal impact on the steady-state permeance when υ is near zero, as shown in Figure S7b. The viscoelastic retardation time (τ_0_) primarily affects the time taken to reach a steady state but does not impact the final permeance (Figure S7c).

**Figure 4:**
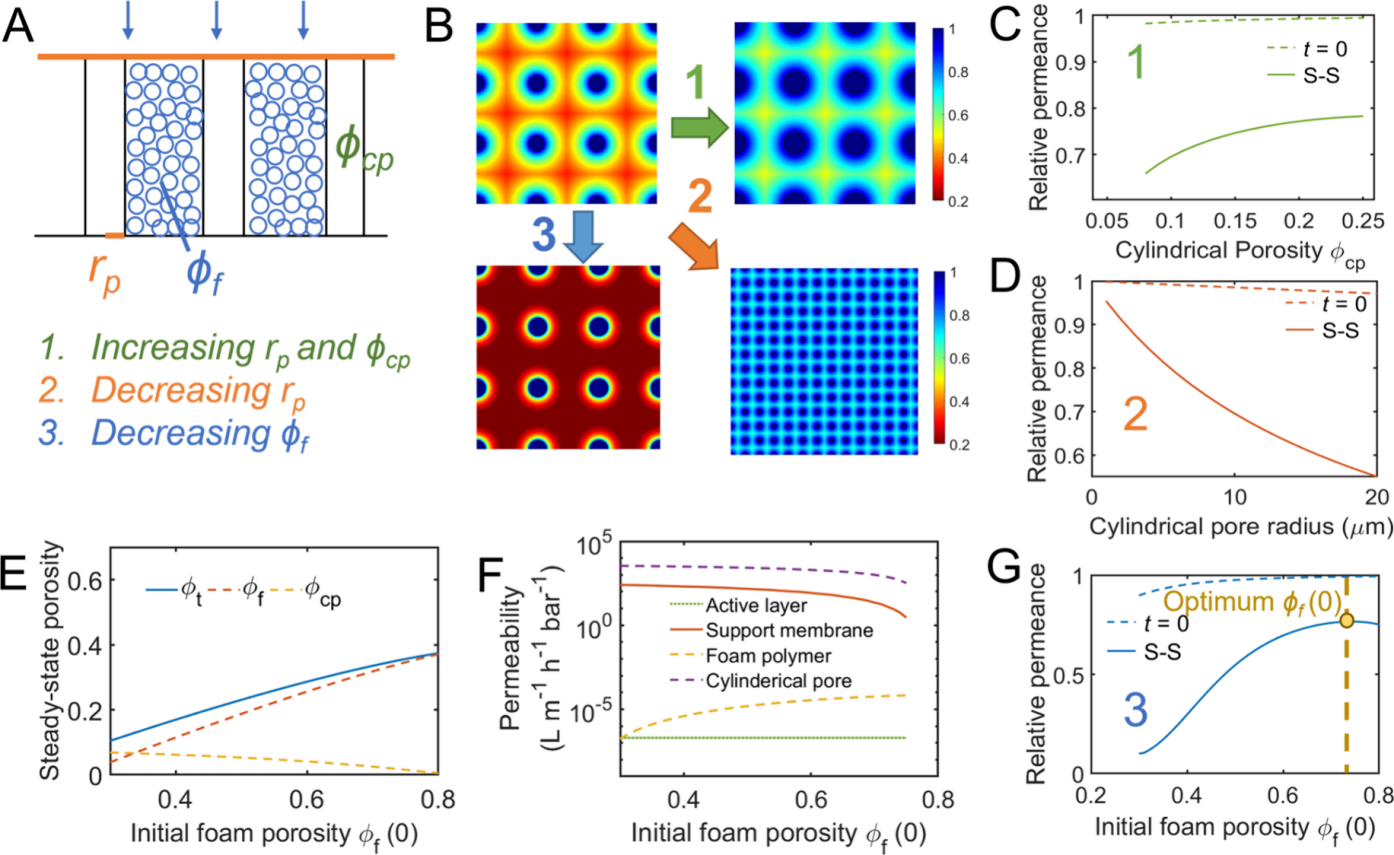
Effect of the initial membrane structure on TFC membrane compaction. (A) Initial morphological parameters investigated: *r*_p_(0), ϕ_cp_(0), and ϕ_f_(0). The baseline values for these parameters: *r*_p_(0) = 10 μm, ϕ_cp_(0) = 0.1, and ϕ_f_(0) = 0.6, given the initial membrane thickness (*L*(0)) = 200 um and the corresponding cylindrical pore density of ∼320/mm^2^. (B) A top-view colormap of the permeance distribution at the baseline value when the membrane reaches steady state. Colormap for scenario 1: increasing *r*_p_(0) while maintaining a constant pore density, resulting in a corresponding increase in ϕ_cp_(0). Colormap for scenario 2: decreasing *r*_p_(0) while keeping ϕ_cp_(0) constant, leading to an increase in pore density. Colormap for scenario 3: decreasing ϕ_f_(0) while keeping other parameters constant. (C) Effect of ϕ_cp_(0) on steady-state permeance (scenario 1), featuring a monotonical increase relationship. (D) Effect of *r*_p_(0) on steady-state permeance (scenario 2), revealing a monotonically decreased relationship. (E) Steady-state ϕ_cp_(*t*_s_), ϕ_f_(*t*_s_), and ϕ_t_(*t*_s_) as functions of ϕ_f_(0). (F) Effect of ϕ_f_(0) on steady-state permeance (scenario 3), explaining the competing effects that lead to an optimal ϕ_f_(0) in the design of support membranes. (G) The steady-state permeability of the foam polymer, cylindrical pores, and the whole support membrane as functions of ϕ_f_(0). The contradictory trends observed in (E) and (F) explain the results presented in (G).

The effect of the initial structure of the support membrane on the compaction is shown in Figure 4. Three primary structural parameters are discussed: cylindrical pore radius (*r*_cp_), cylindrical pore fraction (ϕ_cp_), and foam porosity (ϕ_f_), which were defined earlier and were also labeled in Figure 4A. The geometric relationship between *r*_cp_ and ϕ_cp_ can be described as follows:
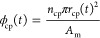
6where *n*_cp_ stands for the number of cylindrical pores in the membrane at a given membrane area *A*_m_. For simplicity, we neglect any effects of the pore size distribution. According to eq 6, when *r*_cp_(0) increases while maintaining a constant pore density *n*/*A*_m_, ϕ_cp_(0) also increases with *r*_cp_(0). Conversely, if ϕ_cp_(0) remains constant, then an increase in *r*_cp_(0) will lead to a decrease in pore density. In Figure 4B, the steady-state permeance colormap for the baseline support structure is shown, using the parameters listed in Table S1, with a transmembrane pressure of 50 bar. We discussed the effects of each structural parameter by considering three scenarios: Scenario 1. Pore density remains constant, while ϕ_cp_(0) varies with *r*_cp_(0). Scenario 2. The initial cylindrical pore fraction ϕ_cp_(0) remains constant, but *r*_cp_(0) changes, leading to the variation in cylindrical pore density. Scenario 3. The initial cylindrical pore fraction ϕ_f_(0) changes, while keeping other parameters constant. For scenario 1 (Figure 4C), both the initial permeance (dashed line) and the steady-state permeance (solid line) monotonically increase with the increase of ϕ_cp_(0) (and consequently *r*_cp_(0)) when the pore density remains constant. In scenario 2 (Figure 4D), both the initial permeance and the steady-state permeance decrease monotonically with the increase of *r*_cp_(0). This finding indicates that support membranes with higher pore densities but smaller “finger-like” pores are preferable compared to supports with sparser “finger-like” pores but larger pore sizes. In addition, it seems that membrane compaction amplifies the initial permeance differences, indicating that structures with higher initial permeances also experience a lower permeance decline.

In scenario 3, the initial porosity of the foam polymer (ϕ_f_(0)) has an interesting impact on membrane compaction, as shown in Figure 4G. Support membranes made from denser foam polymers (corresponding to smaller initial ϕ_f_(0) values) exhibit slightly smaller initial permeances, but their dependence on ϕ_f_(0) is not significant (dashed line). Upon compaction at 50 bar, the flux decline (difference between solid-line and dashed line) of the initially denser membranes is more pronounced, even though the denser foam polymers are mechanically stronger and experience less compressive strain at the steady state (Figure S8). Moreover, the steady-state permeance initially increased with ϕ_f_(0), then reached an optimum ϕ_f_(0) value around 0.73, and subsequently started to decrease with the further increase of ϕ_f_(0). To understand the trends, we plotted the steady-state ϕ_cp_(*t*_s_), ϕ_f_(*t*_s_), and ϕ_t_(*t*_s_) as the function of the ϕ_f_(0) in Figure 4E, along with the corresponding steady-state permeabilities in Figure 4F. On one hand, a larger initial foam porosity ϕ_f_(0) always leads to a higher steady-state ϕ_f_(*t*_s_) and consequently a higher foam permeability. On the other hand, a larger initial ϕ_f_(0) results in a softer membrane that exhibits larger compressive strains, leading to a decrease in cylindrical pore fractions (ϕ_cp_(*t*_s_)) (and consequently the mean pore size *r*_cp_) in the membrane (Figure 4E). This, in turn, lengthens transport pathways to reach the cylindrical pore in the foam polymer and reduces the permeability of the cylindrical pores (Figure 4F). The combined effects give rise to the existence of an optimum ϕ_f_ of (0). This optimum depends on multiple factors related to the compaction, such as the creep compliance of the original dense polymer (*D*_kv_), the transmembrane pressure (Δ*P*), and the Poisson’s ratio (υ) of the foam, which their increment will result in smaller cylindrical pore fractions (ϕ_cp_(*t*_s_)). Support membranes with a higher ϕ_cp_(0) can shift the optimum ϕ_f_(0) toward higher values. Figure S9 examined the optimum ϕ_f_(0) at different Δ*P*. The calculation of optimum ϕ_f_(0) could guide the design and optimization of support membrane structures, especially for TFC membranes intended for different pressure ranges and applications.

Figure S10 presented the comparison of F-only support structures, on the effect of ϕ_f_(0). Membranes with F-only structures showed a lower initial flux and greater flux decline than their counterpart (C + F) in Figure 4G. Both initial flux and final flux increase monotonically with ϕ_f_(0), suggesting the use of a highly porous foam polymer in the support membranes as long as their strength can meet the requirements of their applications. The impacts of the other parameters such as the active layer thickness (*h*) and support thickness are presented in Figure S11. The flux decline supporting membranes with C + F structures will not be impacted by the support membrane thickness due to the existence of the cylindrical pores, but the F-only structures exhibit greater flux declines with the increase of the support membrane thickness.

**Figure 5:**
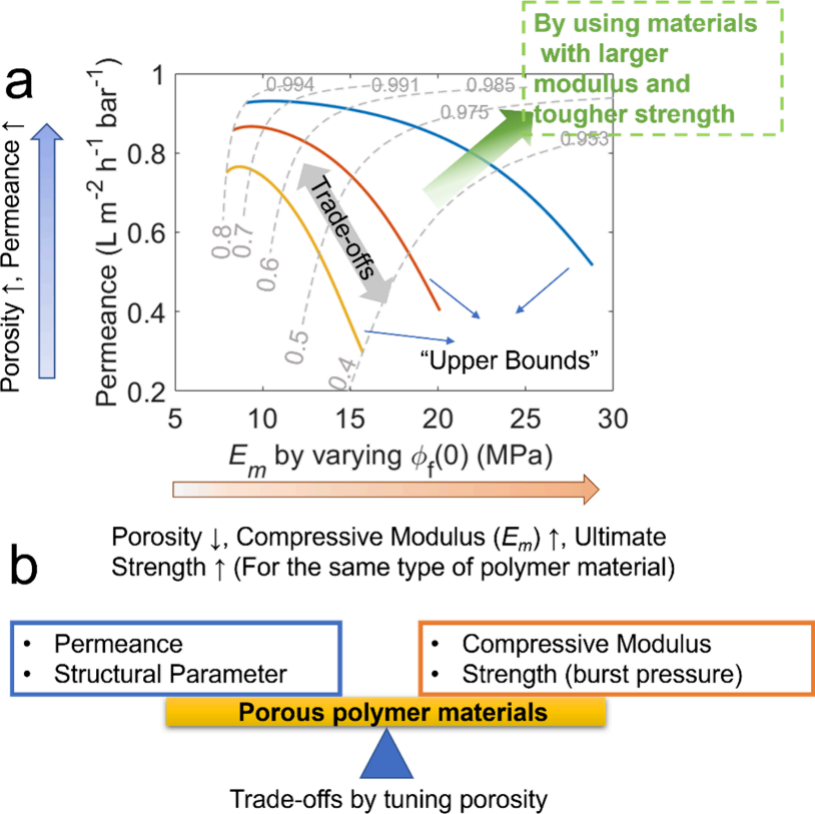
Trade-off relationship between mechanical properties and transport properties of TFC membranes by varying the foam porosity for the same polymer material. (a) The “trade-off plot” of TFC membranes with C + F structures illustrates the relationship between compressive modulus (*E*_m_) and TFC permeance for a specific type of polymer support (represented by a specific *D*_kv_ value). The colored lines represent different *D*_kv_ values: *D*_kv_ = 2.5 × 10^–8^ Pa^–1^ (yellow), *D*_kv_ = 3.75 × 10^–8^ Pa^–1^ (red), *D*_kv_ = 5.0 × 10^–8^ Pa^–1^ (blue). The gray-dashed lines represent isofoam porosity lines ranging from 0.8 to 0.4. The original permeance values are marked on the right side of these lines. Increasing steady-state *E*_m_ is achieved by decreasing the initial foam porosity of the support membrane, indicating that increasing the modulus of the TFC leads to less compaction but results in a higher loss in the steady-state permeance as observed from the intersection points between the gray-dashed lines and the colored lines). (b) Implications of tuning foam porosity for the same polymer support material on the other transport and mechanical properties, assuming the open-cellular pore interconnectivity, pore surface hydrophilicities remain constant. These implications are crucial for various applications, including brine dewatering and beyond.

After investigating the influences of different individual mechanical and morphological parameters, we aimed to gain a deeper understanding of the trade-off relationship between modulus and transport properties when tuning the support porosity of TFC membranes. Figure 5a shows the results using three different polymer compliance (*D*_kv_) values (representing three different polymer materials) and demonstrates the trade-off relationship between steady-state membrane modulus (*E*_m_, *E*_m_*=* 1/*D*_m_(*t*_ss_)) and permeance by varying their initial foam polymer porosity (ϕ_f_(0)), plotted in solid-colored lines, at a transmembrane pressure of 50 bar. To provide a visual reference, we include gray dashed lines representing iso-porosity lines for polymers with different *D*_kv_ values but the same initial ϕ_f_(0). The initial precompaction permeances corresponding to each ϕ_f_(0) are marked on the right side of the iso-porosity lines. For each solid-colored line (which represents a certain kind of polymer), a larger modulus can be achieved by reducing ϕ_f_(0), but this comes at the expense of steady-state permeance after compaction. This result indicates that membranes with higher support foam porosities are preferred, as they experience less flux decline, despite the compaction being more pronounced. In addition to the C + F support structures mentioned above, we also explored the foam-only polymer case, which is presented in Figure S12. A similar trade-off between steady-state modulus and permeance by varying ϕ_f_(0) can also be discovered, which indicates the existence of this trade-off among different support structures.

However, the compressive strength of the porous polymer may act as a limiting factor for a larger ϕ_f_(0). Previous studies on PVDF ultrafiltration membranes prepared using thermally induced phase separations (TIPs) have shown an upper-bound relationship between permeability and tensile strength for such membranes.^59^ Although no theoretical models currently connect modulus with strength (or polymer porosity with strength), experiments on polylactic scaffolds have shown a positive correlation (*R*^2^ = 0.6379) between compressive strength and solid fractions (1 – ϕ_f_(0)).^31^ Additionally, the membrane structural parameter (*S*) widely used in forward osmosis,^60,61^ pressure-retard osmosis (PRO) or osmotically assisted reverse osmosis (OARO), is also negatively related to foam porosity, and minimizing *S* by increasing the support porosity also involves a trade-off with ultimate strength.^46,53^ In the future, understanding the impact of tuning support membrane polymer porosity toward the trade-off relationship between mechanical properties (such as compressive modulus and strength) and transport properties (such as permeance and structural parameter) would be highly beneficial in future applications employing TFC membranes (Figure 5b).

Thus, we have developed a quantitative model to understand the compaction mechanism of TFC membranes, establishing a triangular relationship among compressive modulus, structural, and permeability parameters. The model effectively captures the flux decline behavior of TFC membranes up to 70 bar and provides mechanistic insights into solvent transport during membrane compaction considering realistic mechanical and structural parameters. Our analysis identifies the increases in the transport resistance at the support surface layer just below the active layer as the major factor contributing to membrane flux decline. Support polymers with a higher compressive modulus (lower compliance), supports with higher densities of “finger-like” pores, and “sponge-like” pores with optimum void fractions will be preferred to mitigate compaction. By tuning the initial foam porosity of the support, a quantifiable trade-off relationship between permeance and modulus can be observed. Based on the findings of our simulation, various approaches can be explored to overcome trade-off relationships that impose upper bounds on water permeance/structural parameters and membrane modulus/strength. For instance, employing stiffer support polymers, incorporating nanofillers into the support polymers,^56^ and employing rational design strategies for foam polymer pore profiles using finite element analysis combined with experimental pore tuning strategies, such as conventional phase separation and 3D-printing techniques.^62^ In addition, advanced characterization technologies regarding membrane compaction should also be implemented to refine compaction modeling, such as ultrasonic time-domain reflectometry (real-time),^63^ electrochemical impedance spectroscopy (real-time),^64^ isolation of the PA layer for nanomechanical testings,^65,66^ and nanoimprint lithography.^29,30^ In summary, the time-dependent modulus–structure–transport relationships developed in this study not only attribute to the development of next-generation TFC membranes for applications such as HPRO, OARO, and PRO but also hold relevance for other TFC-like structures in different fields. For instance, the findings may inform the design of scaffolds with biomimetic epidermal coating layers for surgical applications^67^ and other thin-film-coated porous materials.

## Supporting Information Available

Model development; model validation and simulation parameters; distribution of the minimum resistance transport pathways; Supporting Figures 1– 13; Supporting Tables 1–3 (PDF)
Supporting Video (MP4)
