# Supplementary material for: Thin-Film Composite Membrane Compaction: Exploring the Interplay among Support Compressive Modulus, Structural Characteristics, and Overall Transport Efficiency
Source: Environ Sci Technol. 2024 Apr 29;58(19):8587–96. doi: 10.1021/acs.est.4c01639 (PMC11097391; doi:10.1021/acs.est.4c01639)
Supplement: Supplementary file 1 — es4c01639_si_001.pdf [file es4c01639_si_001.pdf]

Supporting Information

for

TFC membrane compaction: Exploring the interplay among support compressive modulus, structural characteristics, and overall transport efficiency

Chunyan Xu[1,2,†], Zhongzhen Wang[3,†], Yuhang Hu[3,4], Yongsheng Chen[2,*]

[1] *School of Resources & Environmental Engineering, Anhui University, Hefei, Anhui, China 230012*

[2] *School of Civil & Environmental Engineering, Georgia Institute of Technology, Atlanta, Georgia 30332-0100, United States*

[3] *School of Chemical & Biomolecular Engineering, Georgia Institute of Technology, Atlanta, Georgia 30332-0100, United States*

[4] *Woodruff School of Mechanical Engineering, Georgia Institute of Technology, Atlanta, Georgia 30332-0100, United States*

[†] These authors contributed equally to this work.

[*] Corresponding Author: yongsheng.chen@ce.gatech.edu

Number of Pages: 31

Number of Figures: 13

Number of Tables: 3

1. Model Development

The changes in the support membrane morphologies due to polymer deformation are believed to be the primary reason for the observed flux decline during compaction.[1-3] Davenport et al.[2] prove that for the dense active layer during compaction, the active layer pore size stays almost the same before and after compaction, as evidenced by positron annihilation lifetime spectroscopy (PALS). Meanwhile, the support membrane undergoes pore collapse during this compaction process, as indicated by the liquid porosimetry and SEM.[2] Thus, if the relationship between membrane flux and the time-dependent support porosity is known, we can estimate the membrane flux. The sections below will provide a detailed derivation for the calculation of membrane-dependent flux.

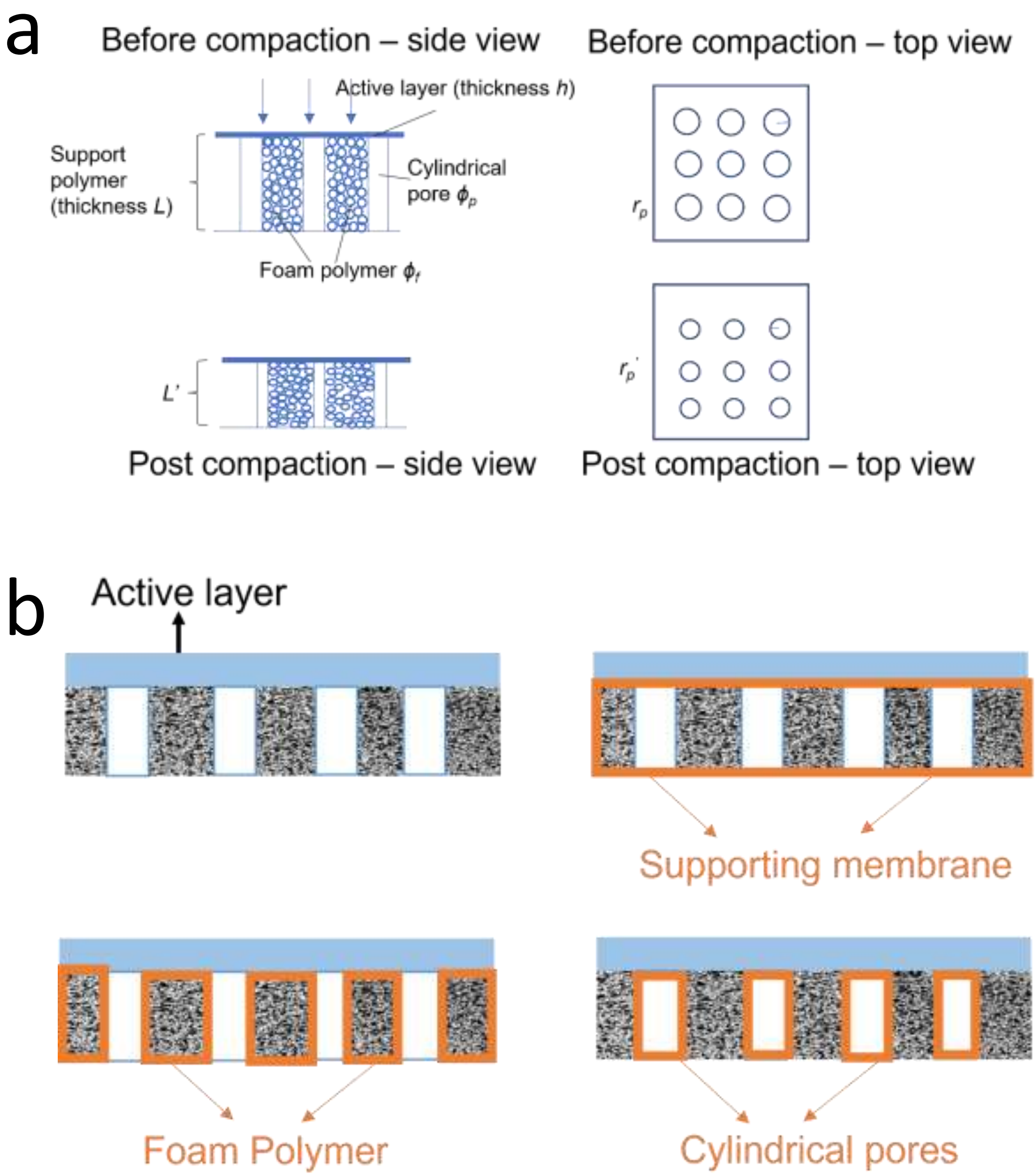

Figure S1. **(a)** Schematic diagram of membrane compaction. **(b)** Scheme of TFC membranes with terminologies of different parts.

1.1 Support membrane structures (Linkage arrow "Compaction" in **Figure 1***)*

As shown in **Figure S1a**, in membrane compaction processes, the external hydraulic pressure difference across the membrane caused the compression of the support membranes, leading to the time-dependent change of the support membrane morphologies, and eventually resulting in the time-dependent change in the intrinsic permeability (and thus flux) of the support membrane. The initial morphology of the support membrane plays an essential role in the whole compaction process, as it not only sets up the initial conditions of the membrane compaction but also affects the subsequent time-dependent creep behavior of the support polymer. Therefore, the first task of compaction modeling is to establish a simplified model of support membrane with proper pore structures matching with SEM observations and realistic, measurable structural parameters.

The structural features of the flat sheet support membranes originated from the phase inversion process. Polymer solutions are cast on glass plates and then transferred to non-solvent such as water, resulting in the precipitation of the polymer and thus membrane formation. Depending on the polymer precipitation rates, two distinct pore structures can form the "sponge-like" pore morphology formed at slower solvent demixing rates, and the "finger-like" morphology formed at higher solvent demixing rates. These two morphologies are not mutually exclusive but co-exist in a support membrane, and their fractions are dependent on the preparation conditions.[6] The water and salt permeability in the "sponge-like" pores is normally smaller compared with the "finger-like" pores.[6] In addition, membranes with fewer "finger-like" pores also have stronger mechanical properties. As a result, in the applications such as reverse osmosis or nanofiltration,

the support membranes are dominated by the "sponge-like" pores, with much smaller fractions (< 0.2) of the "finger-like" region,[6] due to the demand of high solute rejections and higher transmembrane pressures, and these structures are also confirmed by SEM observations of commercial RO and NF membranes.

Therefore, in this work, we proposed to model the support membrane morphology as a uniform flat-sheet foam ("sponge-like") polymer with open cellular pores, at thickness (L). The porosity of the foam polymer (excluding any "finger-like" pores) is ϕ_f. The "finger-like" pores are modeled as cylindrical pores perpendicular to the membrane surface penetrating through the full thickness of the support foam polymer and are uniformly distributed. The locations of these terminologies are presented in **Figure S1b**. The fraction of all the cylindrical pores to the whole volume of the support is ϕ_{cp}. The radius of the cylindrical pores is r. Atop the support membrane is the active layer of the membrane, with thickness h. The following mathematical relationship can be derived (Equations 1-4).

The total mass of the solid polymer contained in the membrane (m_{sp}), the mass of the foam polymer (m_{fp}), and the mass of the membrane should be the same (m_m), and mass conservation is always valid during the whole compaction processes:

$$m_{sp} = m_{fp} = m_m \quad (1)$$

$$\rho_{sp} V_{sp} = \rho_{fp}(t) V_{fp}(t) = \rho_m(t) V_m(t) \quad (2)$$

Where ρ and V denote the corresponding density and the volume, respectively. According to the porosity defined above:

$$\phi_f(t) = 1 - \frac{V_{sp}}{V_{fp}(t)} = 1 - \frac{\rho_{fp}(t)}{\rho_{sp}} \tag{3}$$

$$\phi_{cp}(t) = 1 - \frac{V_{fp}(t)}{V_m(t)} \tag{4}$$

Note that these equations are valid at all times during compaction, indicating that $\phi_f(t)$, $\phi_{cp}(t)$, V_{sp}(t), V_{fp}(t), and V_m(t) also obeys the above relationships at all *t* values.

In this work, we neglect all the shear stress among the active layer, support membrane, and the supporting mesh at the back of the support membrane. Only the normal stress induced by the transmembrane pressure exists within the supporting membrane. Upon compaction, the cellular foam polymer undergoes compressive strain in the same direction as the compressive stress. Meanwhile, the cellular foam polymer expands laterally in the planar direction and occupies the volume of the cylindrical pores at a Poisson ratio v. The real-time $\rho_{\mathrm{fp}}(t)$ can be derived in Equations 5 to 10:

$$\rho_{fp}(0)V_{fp}(0) = \rho_{fp}(t)V_{fp}(t) \tag{5}$$

$$\frac{V_{fp}(t)}{V_{fp}(0)} = \frac{\pi(r + \Delta r)^2(L + \Delta L)}{\pi r^2 L} \tag{6}$$

$$\frac{V_{fp}(t)}{V_{fp}(0)} = \left(1 + \frac{\Delta r}{r}\right)^2 (1 + \frac{\Delta L}{L}) \tag{7}$$

According to the definition of the Poisson's ratio, when the strain is not small and assuming a constant Poisson's ratio:

$$\frac{\Delta r}{r} = \left(1 + \frac{\Delta L}{L}\right)^{-\nu} - 1 \tag{8}$$

Substituting equation 8 into 7 yields:

$$\frac{V_{fp}(t)}{V_{fp}(0)} = \left(1 + \frac{\Delta L}{L}\right)^{1-2\nu} = [1 - \varepsilon(t)]^{1-2\nu} \tag{9}$$

Rearranging equation 5 and substituting equation 9 into equation 5, we have

$$\rho_{fp}(t) = \frac{\rho_{fp}(0)}{[1 - \varepsilon(t)]^{1-2\nu}} \tag{10}$$

Dividing both sides by ρ_{sp} yields:[7]

$$\frac{\rho_{fp}(t)}{\rho_{sp}} = \frac{\rho_{fp}(0)}{\rho_{sp}} \frac{1}{[1 - \varepsilon(t)]^{1-2\nu}} \tag{11}$$

Since $\phi_f(0)$ is a measurable quantity, substituting equation 11 into equation 3, the relationship between real-time porosity of the foam ($\phi_f(t)$) and the strain of the foam ($\varepsilon(t)$) is revealed:

$$\frac{\rho_{fp}(t)}{\rho_{sp}} = 1 - \phi_f(t) = [1 - \phi_f(0)] \frac{1}{[1 - \varepsilon(t)]^{1-2\nu}} \tag{12}$$

The mean pore size, area pore density and the pore size distribution can be related to $\phi_{cp}(t)$ as follows:

$$\varphi_{cp}(t) = \frac{A_{cp}(t)}{A_m} \tag{13}$$

$$A_{cp}(t) = \sum_{i=1}^{n} \pi r_i(t)^2 \tag{14}$$

A_{cp} is the total area of the cylindrical pores, A_m is the membrane area. r_i obeys a certain distribution type, such as normal distribution, gamma distribution, etc. n is the number of cylindrical pores. Assuming the number of cylindrical pores is constant during the compaction process, the reduction in the ϕ_{cp} with time will only result in a shrinkage of the pore radius r_i. Details of generating the membrane sample randomly distributed pores by using the above for calculation can be found in the supporting information.

1.2 Viscoelastic modeling of support membranes (Linkage arrow "Viscoelasticity" in Figure 1)

Upon compaction, the polymer exhibited time-dependent compressive strain, known as the viscoelastic behavior. The compressive strain leads to the change in the polymer morphology, such as the cellular pore deformation and the lateral expansion of the foam polymer (in the planar direction in **Figure S1a**), which makes porosities $\phi_f(t)$, $\phi_{cp}(t)$ as time-dependent functions. Therefore, the purpose of this section is to develop a stress-strain-morphology relationship that can be used to derive the expressions of $\phi_f(t)$, and $\phi_{cp}(t)$ for the permeability calculations in **Supporting Information section 1.3**.

The compressive strain of a solid polymer (porosity equals zero), $\varepsilon_{polymer}(t)$, can be normalized by the compressive stress σ as a quantity called tensile creep compliance, $D_{sp}(t)$:

$$D_{sp}(t) = \varepsilon_{sp}(t)/\sigma \qquad (15)$$

The transient tensile creep compliance of the solid polymer (D_{sp}) can be calculated by the combination of a Hookean spring, a Kelvin-Voigt element, and a dashpot:[8]

$$D_{sp}(t) = D_0 + D_{KV}\left[1 - \exp\left(-\frac{t}{\tau_{bur,0}}\right)\right] + \frac{t}{\eta_0} \qquad (16)$$

where D_0 is the compliance of the Hookean spring part, D_{KV} represents the compliance of the Kelvin–Voigt element, $\tau_{bur,0}$ is the retardation time, and η_0 represents the viscosity of the material. For membrane polymers, D_0 is significantly smaller than D_{kv} and given by the time scale of membrane compaction (normally less than 10^5 s). Both the first term and the last term in equation 16 can be neglected. This indicates that a Kelvin-Voigt element is sufficient to capture the main feature of polymer creep behavior during membrane compaction:

$$D_{sp}(t) = D_{KV}\left[1 - \exp\left(-\frac{t}{\tau_{bur,0}}\right)\right] \tag{17}$$

For open-cell foam polymers, previous works have shown that the modulus E_{fp} of such foam materials can be related to the modulus of the solid polymer (E_{sp}) as:

$$\frac{E_{fp}(t)}{E_{sp}} = C_1\left(\frac{\rho_{fp}(t)}{\rho_{sp}}\right)^2 \tag{18}$$

Similarly, for the creep compliance of cellular foam polymer (D_{fp}), since D_{fp} is the inverse of E_{fp}[9, 10], now we have the compliances relationship between the foam polymer and solid polymer:

$$D_{fp}(t) = D_{sp}(t) * \left(\frac{\rho_{fp}(t)}{\rho_{sp}}\right)^{-2} / C_1 \tag{19}$$

where C_1 is a constant with a scale of 10^0. Hence we assumed C_1=1 in our simulation. Substituting equation 12 into the above equation gives:

$$D_{fp}(t) = D_{sp}(t) * \left[1 - \varphi_f(0)\right]^{-2}[1 - \varepsilon(t)]^{2-4\nu} \tag{20}$$

From the definition of creep compliance, we have

$$D_{fp}(t) = \varepsilon(t)/\sigma \tag{21}$$

Substituting equation 21 to 20, we can now solve for the real-time strain of the cellular foam polymer by known quantities from equation 22.

$$\frac{\varepsilon(t)}{\sigma} = D_{sp}(t) * \left[1 - \phi_f(0)\right]^{-2}[1 - \varepsilon(t)]^{2-4\nu} \tag{22}$$

Once $\varepsilon(t)$ is known, $D_{fp}(t)$, $\rho_{fp}(t)$,$\phi_f(t)$ can be calculated accordingly using equation 19, 12 and 12, respectively, which will be useful for the next step to support permeability and

membrane flux calculations. The last step is to derive the expression of ϕ_{cp} (t). Recall equation 4:

$$\phi_{cp}(t) = 1 - \frac{V_{fp}(t)}{V_m(t)} = 1 - \frac{V_{fp}(t)/V_m(0)}{V_m(t)/V_m(0)} \tag{23}$$

$$\phi_{cp}(0) = 1 - V_{fp}(0)/V_m(0) \tag{24}$$

Rearranging equation 24 and substituting it into equation 23 gives:

$$\phi_{cp}(t) = 1 - \frac{V_{fp}(t)/V_{fp}(0) * [1 - \phi_{cp}(0)]}{V_m(t)/V_m(0)} \tag{25}$$

For the volume ratio of the membrane, since the area of the membrane is a constant, we have:

$$\frac{V_m(t)}{V_m(0)} = \frac{L_m(t)}{L_m(0)} = 1 - \varepsilon_m(t) = 1 - D_m(t)\sigma \tag{26}$$

$L_m(0)$ is the membrane support thickness at the initial condition, and $L_m(t)$ is the real-time thickness. ε_m (t) is the real-time strain of the membrane, and D_m (t) is the overall compliance of the membrane. Handge[8] derived the relationship between D_m (t) and D_{cp} (t) as in equation 27, assuming the transport path (cylindrical macrovoid pores) weakens the modulus of the membrane linearly:

$$D_m(t) = \frac{D_{fp}(t)}{1 - \phi_{cp}(0)(\tau_m - 1)/\tau_m} \tag{27}$$

Where τ_m is the tortuosity of the support membrane. In this paper, we assume the tortuosity is only contributed by the macro scale cylindrical pores. If the pores are straight, $\tau_m = 1$ and Eq. 27 simplifies to D_m (t) $=D_{fp}$ (t). Also, $\varepsilon_m(t) = \varepsilon(t)$.

For the volume ratio of the cellular foam polymer ϕ_{cp}, we previously calculated it in equation 25. Substituting equation 26 and 27 into equation 25, eventually it gives:

$$\phi_{cp}(t) = 1 - \frac{[1-\phi_{cp}(0)]\left[1-D_{fp}(t)\sigma\right]^{1-2\nu}}{1-D_m(t)\sigma} \qquad (28)$$

Note that σ here denotes the compressive stress inside the foam polymer membranes, but not the transmembrane pressure, ΔP. If ϕ_{cp} is small enough, $\sigma \approx \Delta P$, where ΔP is the transmembrane pressure. If ϕ_{cp} is not small, its relationship with ΔP can be derived from the force balance analysis as $\sigma = \Delta P/(1 - \phi_{cp})$. The influence of different support pore tortuosity under TFC membrane compaction by a transmembrane pressure Δp is shown in **Figures S2a** and **S2b**.[8] For TFC membranes, as the transmembrane pressure (TMP) is Δp, the top surface of the whole TFC membrane will be compressed by a force of $\Delta p*A$, where A is the membrane area. Different from the polymer support alone, for example, UF membrane in **Figure** Error! Reference source not found.**c**, the transmembrane pressure $\Delta P = \Delta p$ on the UF membrane surface. For the support membrane of the TFC membrane, if the total area of TFC membrane surface is A, then the area of surface support will be A*(1- ϕ_{cp}). To maintain force balance, $\Delta P*A\ (1- \phi_{cp}) = \Delta p\ *A$, thus, the pressure exerted on the support membrane polymer (ΔP) will be $\Delta p/\ (1- \phi_{cp})$. An illustration is provided inError! Reference source not found.**d**. By incorporating the above expression, Equation 28 has now become an implicit equation. Thus, we used the iterative method to solve for $\phi_{cp}\,(t)$ and $\phi_f\,(t)$, using the value from the previous step as the initial value.

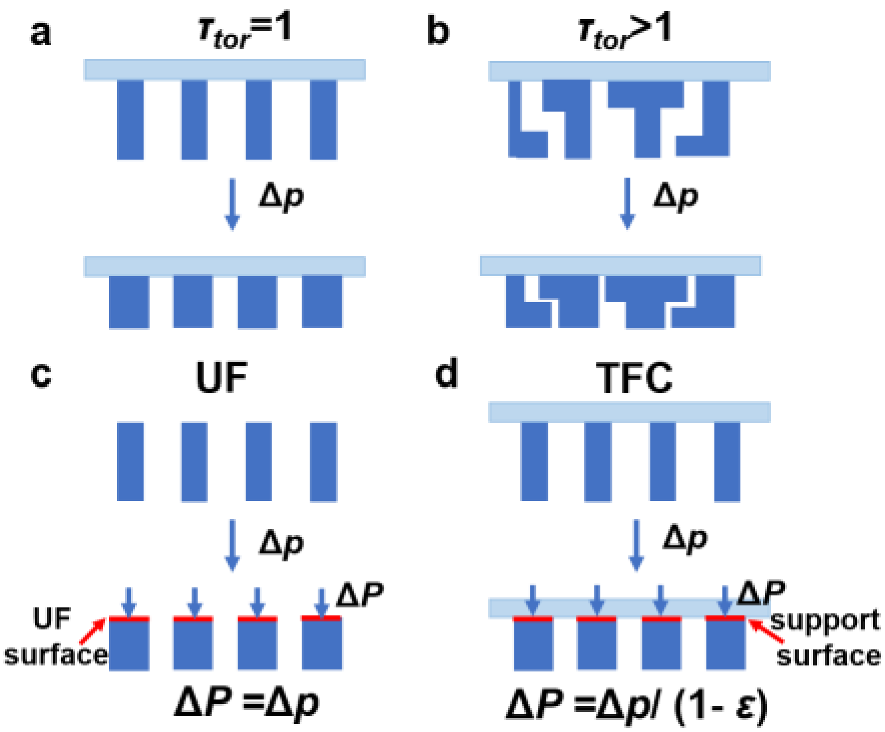

Figure S2. Influence of support membrane pore tortuosity (τ_{tor}) on TFC membrane compaction by a transmembrane pressure, Δp: **(a)** perpendicular pores τ_{tor} =1, and **(b)** non-perpendicular pores τ_{tor} >1. Comparison of the transmembrane pressure on the porous polymer membrane surface (ΔP) with and without thin film active layer: **(c)** transmembrane pressure exerted on ultrafiltration (UF) membrane $\Delta P = \Delta p$, and **(d)** transmembrane pressure exerted on TFC support membrane $\Delta P = \Delta p/(1-\phi_{\mathrm{cp}})$, where ϕ_{cp} represents the cylindrical pore fraction of TFC support membrane.

1.3 Support membrane permeability calculations

Once the time-dependent porosities $\phi_f(t)$ and $\phi_{cp}(t)$ are known, the permeabilities of water can be calculated. The permeability for a specific cylindrical pore i with pore radius r_i may be estimated by the Hagen Poiseuille equation:

$$P_{cp_i}(t) = \frac{r_i(t)^2}{8\mu} \tag{29}$$

The permeabilities of the cellular foam polymer can be calculated from the Brace's

equation:[11]

$$P_{fp}(t) = A_{fp} d_{fp}^2 \phi_f(t)^3 \tag{30}$$

Where d_{fp} is the average cell diameter, and A_{fp} is an empirical constant. In practice, d_{fp} can be determined from the porosimetry measurements, and the coefficient A_{fp} can be calculated by the measurement using a pure foam polymer. Both the scales of the P_{cpi} (t) and P_{fp} (t) are significantly larger than the permeability of the polyamide layer (P_a).

1.4 Water and solute flux calculations by resistance-in-series modeling and Monte Carlo simulations (Linkage arrow "Monte Carlo" in Figure 1)

Once the membrane morphology and the permeability of different membrane parts are known, the next step is to evaluate the water flux of the membrane. Water molecules must transport across the active layer of the membrane first, and then the path through either the foam polymer part, the cylindrical pore part, or both parts, before reaching the permeate side. The resistance-in-series approach treats one transport media with uniform transport property (e.g., water permeability, heat conductivity) as one resistor, and the total transport resistance as the summation of each transport resistance along a transport pathway. Thus, for a given water probe I placed on the surface of the membrane, its path-dependent transport resistance can be calculated by knowing the transport resistance within each part of the membrane. The resistance is determined by the pathway length and the permeability of each part of the membrane. In this work, the following three parts of the membrane are involved: 1. the transport pathway in the active layer, with a length of h_{Ai}; 2. the transport pathway inside the foam polymer of the support membrane, with a length of h_{fi}; 3. the transport pathway in the cylindrical pores of the support membrane, with a length of h_{cpi}.

The water transport resistance in the active layer can be expressed as:

$$R_{AW} = \frac{h_{Ai}}{P_{Ai}} \quad (31)$$

$$R_{FW} = \frac{h_{fi}}{P_{fi}} \quad (32)$$

$$R_{CPW} = \frac{h_{cpi}}{P_{cpi}} \quad (33)$$

Where R_{wi} is the resistance of the water probe i, R_{wi} *is* the total resistance, and J_{wi} is the local water flux of the water probe i, h_{Ai}, h_{Fi}, and h_{CPi} are minimum resistance transport lengths in the active layer, foam polymer, and the cylindrical polymer, respectively. P_{Ai}, P_{Fi}, and P_{CPi} are solvent permeabilities in the active layer, foam polymer, and cylindrical polymer, respectively. The total resistance (R_{wi}) and the water flux (J_{Wi}) can then be expressed as:

$$R_{Wi} = \left(\frac{h_{Ai}}{P_{Ai}} + \frac{h_{Fi}}{P_{Fi}} + \frac{h_{CPi}}{P_{CPi}}\right) \quad (34)$$

$$J_{Wi} = \frac{\Delta P}{R_{Wi}} \quad (35)$$

In this work, we assume that the water probe i will take the transport pathway with the lowest transport resistance (MRTP). Thus, the transport lengths h_{Ai}, h_{Fi}, and h_{CPi} will be determined mathematically by finding the minimum value of the transport resistance. For every single water probe, four adjacent support surface pores are taken into consideration (**Figure S3a**) in order to determine the shortest path of the water transport, and it is assumed that the water probe will take the path with the least resistance (shortest path). This calculation was repeated 10,000 times on a periodic cell to generate a statistically reliable mean value of the water flux, by the summation of

the water flux of all the water probes and dividing the number of points used. **Figure S3b** shows the 3D model of one period cell with one support pore (pore size r_1) in the center of the interface between the active layer and the support layer surface. One sample water probe i enters from the top surface of the active layer.

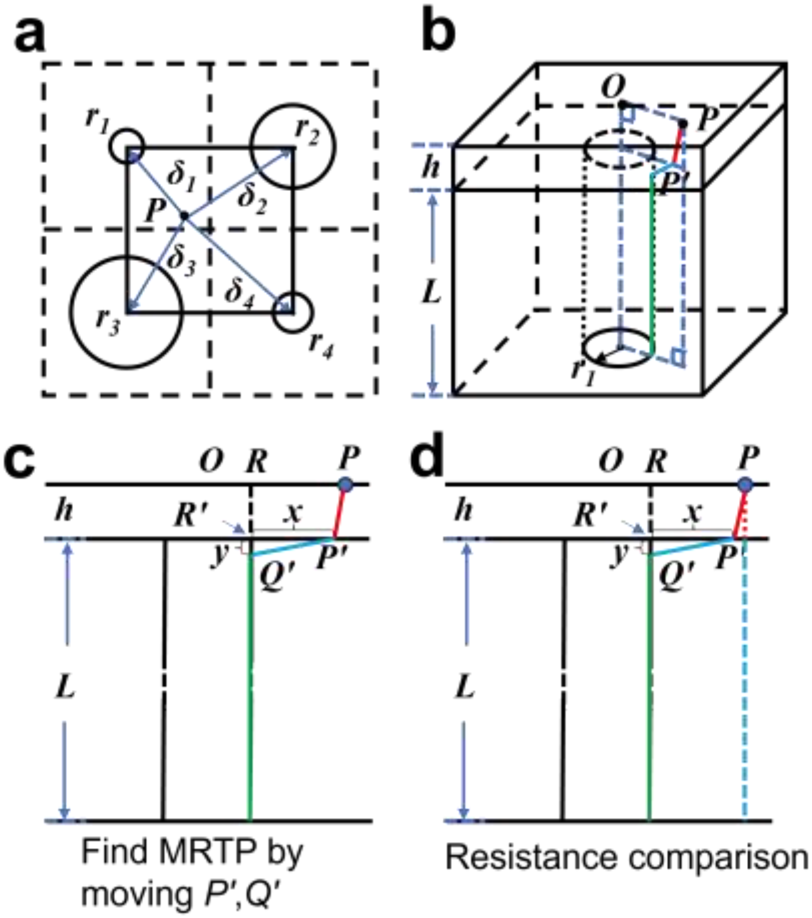

Figure S3. Schematic of the geometry used in the water transport path calculation. (a) TFC membrane surface top view. The dashed lines show the edges of four sample squares with four pores (random pore sizes r_1, r_2, r_3, and r_4) located in the center of each sample square. Water probes that fall within the solid square area (for example, Probe P) need to calculate their shortest water transport pathways (δ_1, δ_2, δ_3, and δ_4) with respect to the 4 pores located at the vertex of the solid square. (b) 3D model of the periodic pore cell with one cylindrical pore in the center with pore size r_1 from (a). One sample water probe P enters from the top surface of the active layer. (c) Cross-sectional view of (b). The determination of MRTP of probe P if P is placed atop the foam polymer (d) Comparison of MRTPs with the straight penetration scenario. Since $P_{cp} >> P_f$, and $L >> h$, the straightly penetrated situation is not common unless P is very far away from the cylindrical pores.

The algorithm of finding the MRTP for a specific MC probe is presented as below. If a MC probe is placed atop of the cylindrical pore, then the MRTP is easy to determine, which is a straight

line perpendicular through the membrane. The resistance is:

$$R_{Wi} = (\frac{h_{Ai}}{P_{Ai}} + \frac{h_{CPi}}{P_{CPi}}) \tag{36}$$

Here, h_{Ai} equals the thickness of the active layer ($h_{Ai} = h$), h_{Cpi} equals the thickness of the support layer ($h_{Cpi} = L$).

However, if the MC probe is placed atop of the foam polymer (**Figure S3b &c**), then the MRTP involves finding the minimum of the objective function (R_{wi}), by optimizing h_{Ai}, h_{cpi} and h_{Fi}. By knowing the distance of the MC probe to the top center of the cylindrical pore (line *PR*, length *d*), the active layer and support thickness and permeabilities, R_{wi} can be expressed as the function of the length of line *P'R'* (*R'* is on the line *P'O'*) in Figure X, with distance *x*, and line *R'Q*, with distance *y*:

$$h_{Ai} = \sqrt{(d-x)^2 + h^2} \tag{37}$$

$$h_{Fi} = \sqrt{x^2 + y^2} \tag{38}$$

$$h_{CPi} = L - y \tag{39}$$

$$R_{Wi} = \frac{\sqrt{(d-x)^2 + h^2}}{P_{Ai}} + \frac{\sqrt{x^2 + y^2}}{P_{Fi}} + \frac{L - y}{P_{CPi}} \tag{40}$$

Then, the MRTP can be found by setting $\partial R_{Wi}/\partial x = 0$ and $\partial R_{Wi}/\partial y = 0$ and solving for *x*, *y*. However, the R_{wi} should be compared with the straightly penetrated case, and the smaller R_{wi}

should be used finally:

$$R_{Wi} = \frac{h_{Ai}}{P_{Ai}} + \frac{h_{Fi}}{P_{Fi}} \tag{41}$$

For the calculation of the overall membrane water flux, we used large numbers of evenly distributed water probes on the periodic box to better estimate the water flux with respect to support membrane morphologies. The water flux of the membrane (J_W) is then determined by the average water flux of all the water probes.

$$J_W = \frac{1}{n}\sum_{i=1}^{n} J_{Wi} \tag{42}$$

Thus, the real-time membrane flux can be solved by using the time-dependent morphology correlations derived in Equation 28.

2. Model validation and simulation Parameters

The simulation parameters are tabulated in **Table S1**, including the references. In this model, D_{kv} and $\tau_{bur,0}$ is selected as the fitting parameters for different types of membranes when validating the model with experimental data. Other parameters are fixed parameters. **Table S2** lists the relationship between the number of Monte Carlo simulation points and the calculation accuracy for the steady-state permeance.

Table S1. All parameters used in fitting NF and RO membrane flux vs time data in **Figure 3a** & **3b**, and the baseline values used for the subsequent compaction modeling. Parameters marked with a red asterisk (*) represent fitting parameters.

**Nomenclature**		**NF-90** Hussain et. al[3]	**SW-30** Davenport et. al[2]	**Baseline value in simulation**	**Units**	**References**
Initial structural parameters (t = 0)						
The volume fraction of the cylindrical pores to the membrane	ϕ_{cp}	0.10	0.10	0.10	-	[2]
Average pore radius of the cylindrical pores	R_{avg}	10	10	10	um	[2]
The porosity of the foam polymer	ϕ_f	0.60	0.60	0.60	-	[2]
The total porosity of the support	ϕ_t	0.64	0.64	0.64	-	$\phi_t = \phi_{cp} + (1- \phi_{cp})\ \phi_f$
Cell diameter of the foam polymer	d_0	100	100	100	nm	Estimated.
Support membrane thickness (including the PET layer)	L	200	200	200	µm	3
The Poisson ratio of the foam polymer	v	0.050	0.050	0.050	-	[7, 12]
Active layer thickness	h	200	200	200	nm	[2]
Mechanical parameters						
Creep compliance of the Keven-Voigt element of the bulk polymer *	D_{kv}*	6.40×10^{-8}	3.30×10^{-8}	5.0×10^{-8}	Pa^{-1}	* Fitting parameter
The viscoelastic response characteristic time *	τ_0 *	1500	200	1800	s	* Fitting parameter
Foam density-modulus correlation parameter	C_1	1	1	1	-	[8]
Permeances and Permeabilities						
The permeance of the active layer	P_a/h	6.0	1.32	1	$L\ m^{-2}\ h^{-1}\ bar^{-1}$	[2, 3]
The permeability coefficient of foam polymer	A_{fp}	1	1	1		Estimated from[7]
External operating parameter						
Transmembrane pressure	ΔP	10, 20, 30	30-110	50	bar	

Table S2. Required numbers of evenly distributed Monte Carlo (MC) probes to accurately assess the relative permeance of the periodic cell with the cylindrical pore at the center in **Figure S3**. The steady-state relative permeances are values calculated using the baseline values listed in **Table S1**. As the number of the MC probes increases, the relative permeance converges. Here, we set the relative permeance values when using 1×10^6 numbers of MC points as the accurate value and calculated the percentage errors of using fewer MC points. Using 10000 MC points per unit cell could effectively control the percentage error of the relative permeance below 0.23%, and therefore, 10000 MC points were selected to evaluate the relative permeance in this work. (Marked as the green bold text in the table)

MC probe points on the periodic cell	Relative permeance	Error %
**36**	0.7333	4.17
**100**	0.7182	2.66
**400**	0.7039	1.24
**1024**	0.6990	0.75
**4096**	0.6952	0.36
**10000**	**0.6938**	**0.22**
**40000**	0.6925	0.10
**1×10^6**	0.6916	0.00

3. Discussions regarding the distribution of the minimum resistance transport pathways (MRTPs) in this work

Table S3. Pore diameter ranges, calculation formulars and possible water permeability ranges for cylindrical pores, foam polymer and active layer.

Part of the TFC membrane	Pore diameter ranges	Pore characterization technique	Permeability calculation formula	Water permeability ranges ($L\ m^{-1}\ h^{-1}\ bar^{-1}$)
**Cylindrical pores (P_{cp})**	1 - 50 um	Cross-sectional SEM[2, 13]	$P_{cp} = \frac{r^2}{8\mu}$	$50 \sim 1.3\times10^5$
**Foam polymer (P_f)**	10-5000 nm	SEM and porosimetry[2]	$P_{fp} = A_{fp} d_{fp}^2 \phi_f$ [3]	$7.8\times10^{-6} \sim 7.8\times10^{-2}$
**Active layer (P_a)**	<= 1nm or nonporous	Positron annihilation spectroscopy[14]	P_a from TFC filtration experiments; assuming 200 nm thickness P_a = Permeance×h	$2\times10^{-7} \sim 1\times10^{-6}$

Table S3 shows the possible permeabilities of the cylindrical pores (P_{cp}), foam polymer (P_f) and active layer (P_a). Based on the characterization techniques regarding their pore sizes, we can now safely argue that in TFC membranes, $P_{cp} >> P_f >> P_a$. Based on the above permeability sequence, we evaluated the minimum resistance transport pathway (MRTP) distributions in **Figure S4**. We find that even under extreme cases, when $P_f = 10\ P_a$, the MRTPs are still located in the "interface layer" atop the foam polymer, which has a thickness comparable to the active layer but significantly thinner than the thickness of the support (**Figure S4e**). When P_f increases the thickness of this layer decreases. (**Figure S4f**)

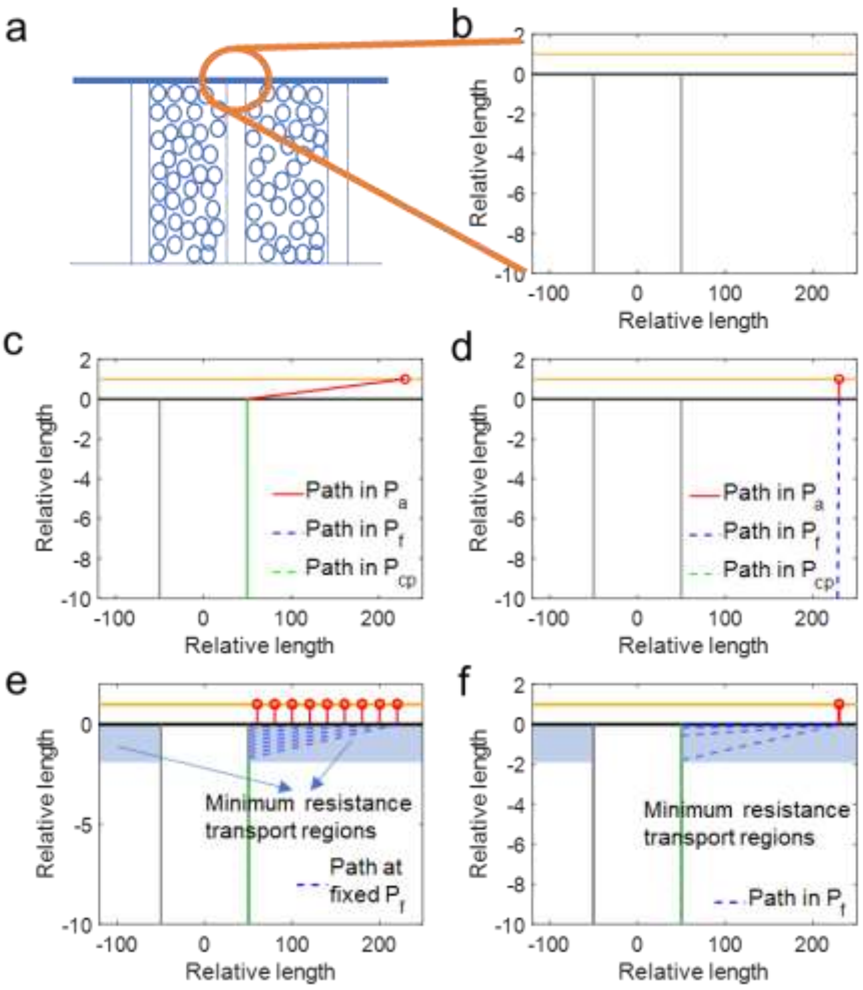

Figure S4. Minimum resistance transport pathways (MRTPs) distributions. **(a)** TFC membrane side-view structure. **(b)** TFC membrane side-view near the active layer – support membrane interface. **(c)** MRTPs locations if $P_{cp} >> P_a = P_f$. The MRTPs will always be in the active layer and the cylindrical pores. **(d)** MRTPs locations if $P_{cp} = P_f >> P_a$. The MRTPs will always be in the active layer and the cylindrical pores. **(e)** The realistic permeability scenario in TFC membranes, where $P_{cp} >> P_f >> P_a$. Here we assume $P_{cp} = 10^7\ P_f = 10^8\ P_a$, which might be the most extreme scenario in a TFC membrane since P_f is only 10 times the value of P_a. It seems that all the MRTPs for different Monte Carlo probes are still located in the thin layer at the top part of the active layer, with thickness comparable to the active layer thickness, which is referred to as the "interface layer" in this work **(f)** The effect of foam polymer permeability on the MRTPs distributions. It seems that the increase in P_f will lead to the MRTPs moving toward the interface of the active layer and the support foam polymer. These results highlighted the importance of the "interface layer" to membrane transport and their vital roles in membrane compaction.

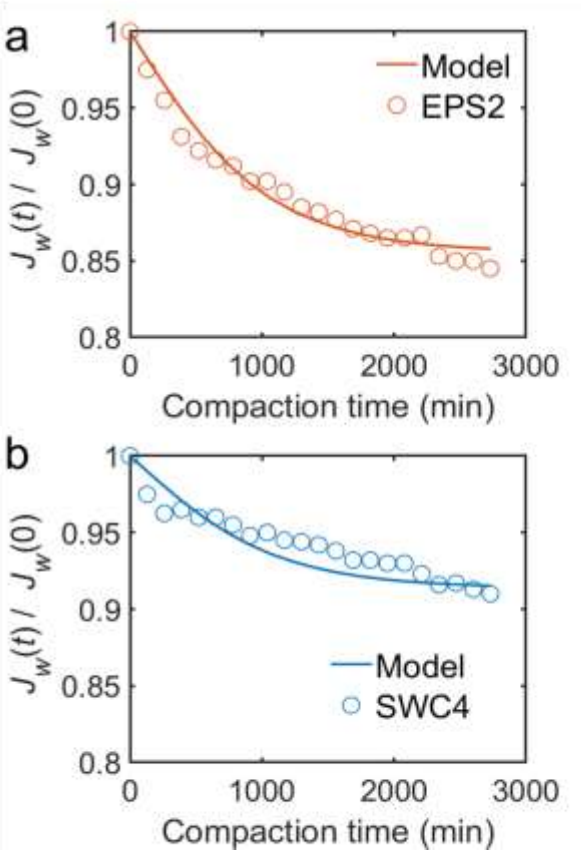

Figure S5. Model fitting of flux decline data at 50 bar *ΔP* by Zhao et. al.[4] **(a)** ESP2 membrane, a brackish water desalination membrane. Parameters from source: ϕ_{cp} = 0.189, L = 27.5 µm, permeance = 3.72 L m^{-2} h^{-1}. Fitting Parameters: D_{kv} = 2.5 × 10^{-8} Pa^{-1}, τ_0 = 600 min. **(b)** SWC4 membrane, a seawater desalination membrane. Parameters from source: ϕ_{cp} = 0.128, L = 40 µm, permeance = 1.42 L m^{-2} h^{-1}. Fitting Parameters: D_{kv} = 2.5 × 10^{-8} Pa^{-1}, τ_0 = 600 min. Other parameters are the same as the baseline values in **Table S1**.

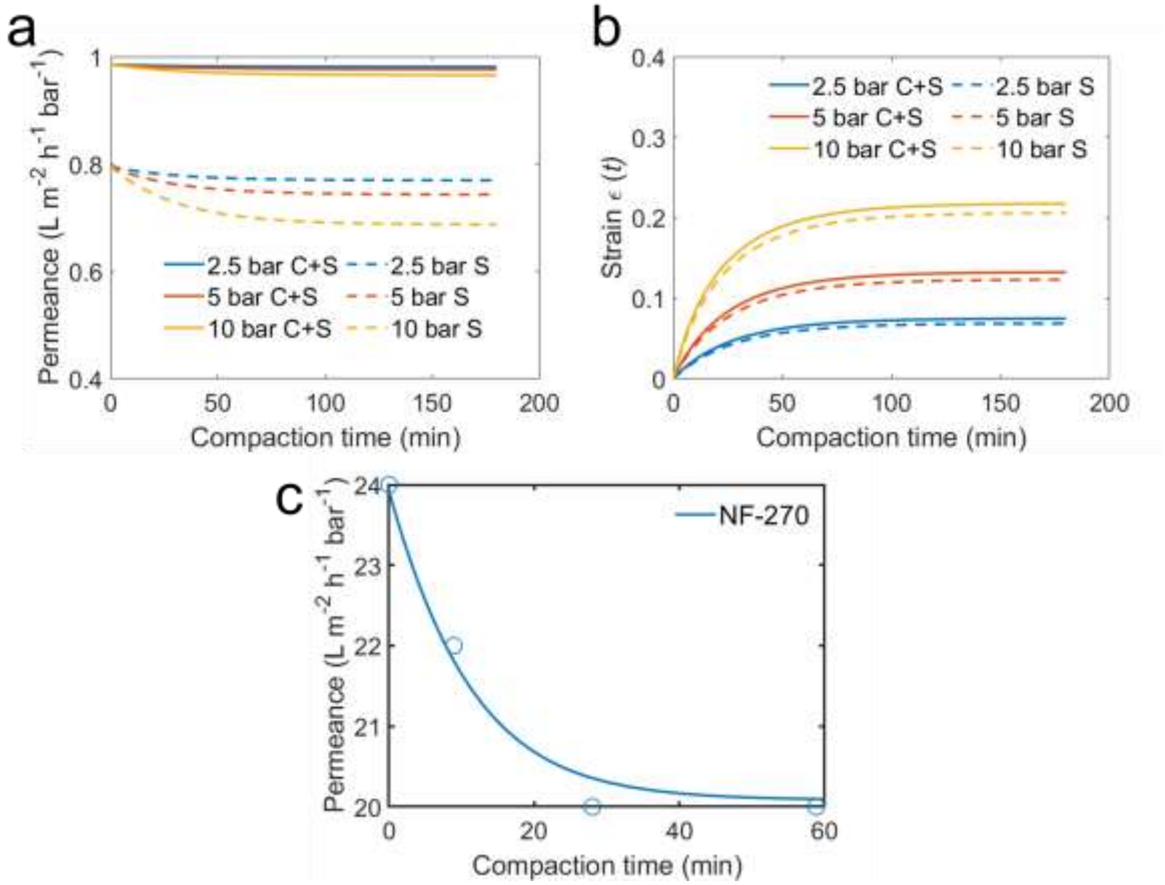

Figure S6. Comparison of model with cylindrical pores and without cylindrical pores (foam-only support), assuming they are made of the same polymer foam. **(a)** Comparison of permeance decline of cylindrical pore plus foam polymer (C+F, in solid lines) and foam support (F, in dashed lines). **(b)** Comparison of the compressive strain of cylindrical pore plus foam polymer (C+F, in solid lines) and foam support (F, in dashed lines). It seems that the strains are similar with or without the cylindrical pores. **(c)** Flux decline fitting of a representative TFC membrane, NF-270, at 15 bar transmembrane pressure. Data source from Semião et al.[5] The fitting parameters are D_{kv} = 5.0×10^{-8} Pa^{-1}, τ_0 = 10 min.

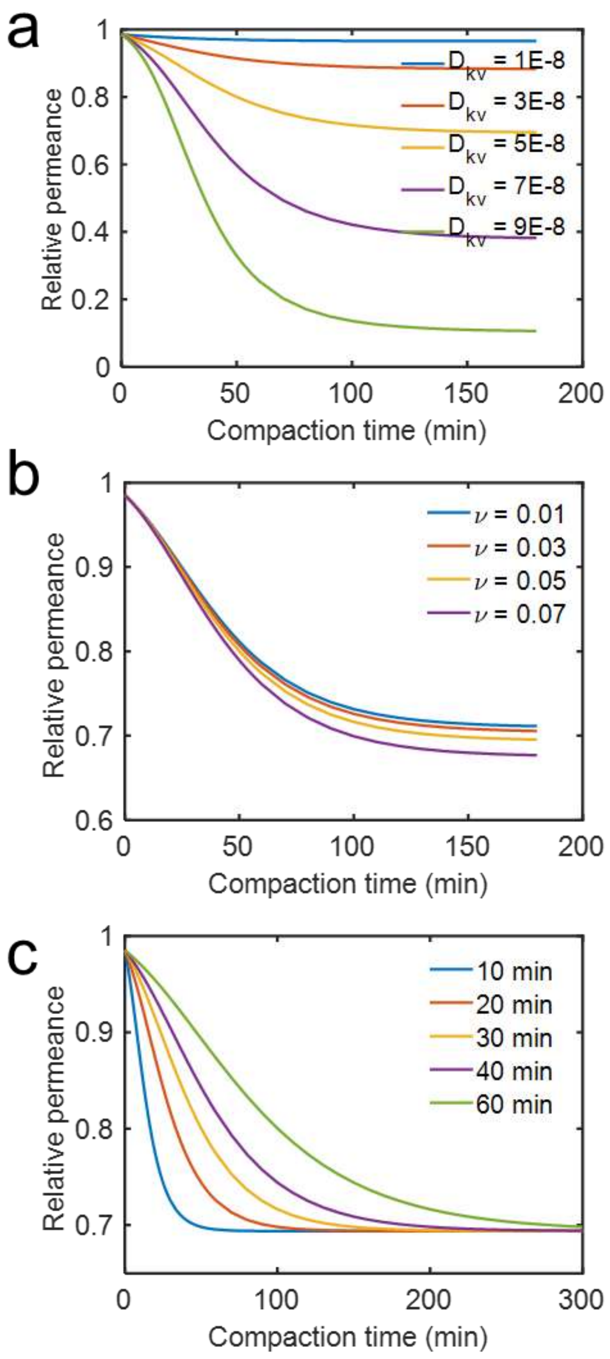

Figure S7. Effect of different mechanical parameters on membrane compaction. **(a)** Effect of creep compliance (D_{kv}) on compaction. **(b)** Effect of Poisson's ratio (v) on compaction. **(c)** Effect of retardation time (τ_0) on compaction.

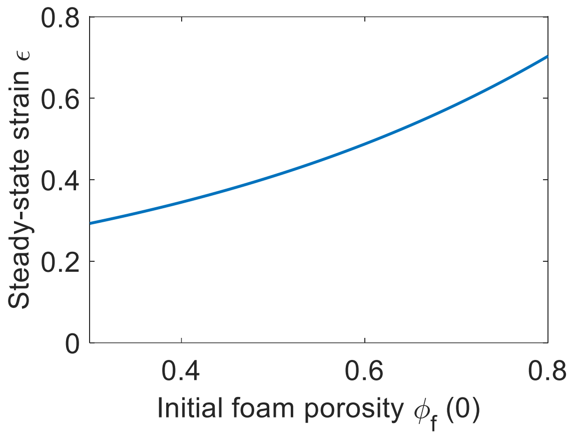

Figure S8. Effect of initial foam porosity on the steady-state strain of the membrane. Apparently, the membranes with higher $\phi_f(0)$ values are more vulnerable to compaction, but they experience less flux decline in **Fig. 4g**.

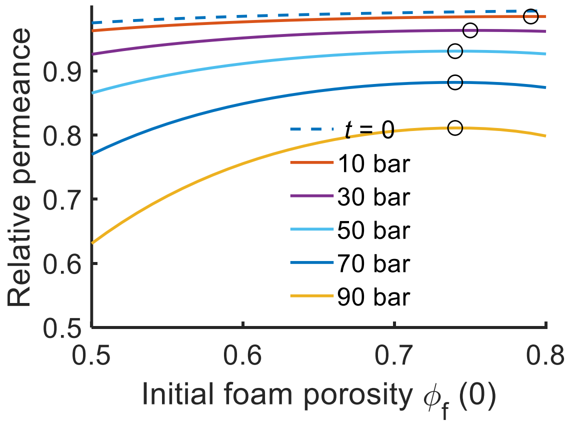

Figure S9. Effect of transmembrane pressure on the optimum $\phi_f(0)$ of the membrane.

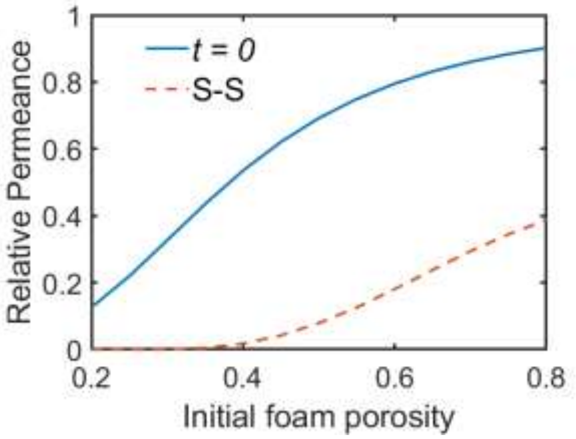

Figure S10. Effect of initial foam porosity on the initial permeance (t = 0) and steady-state permeance of TFC membrane with foam-only supports. (Compare with **Figure 4g**)

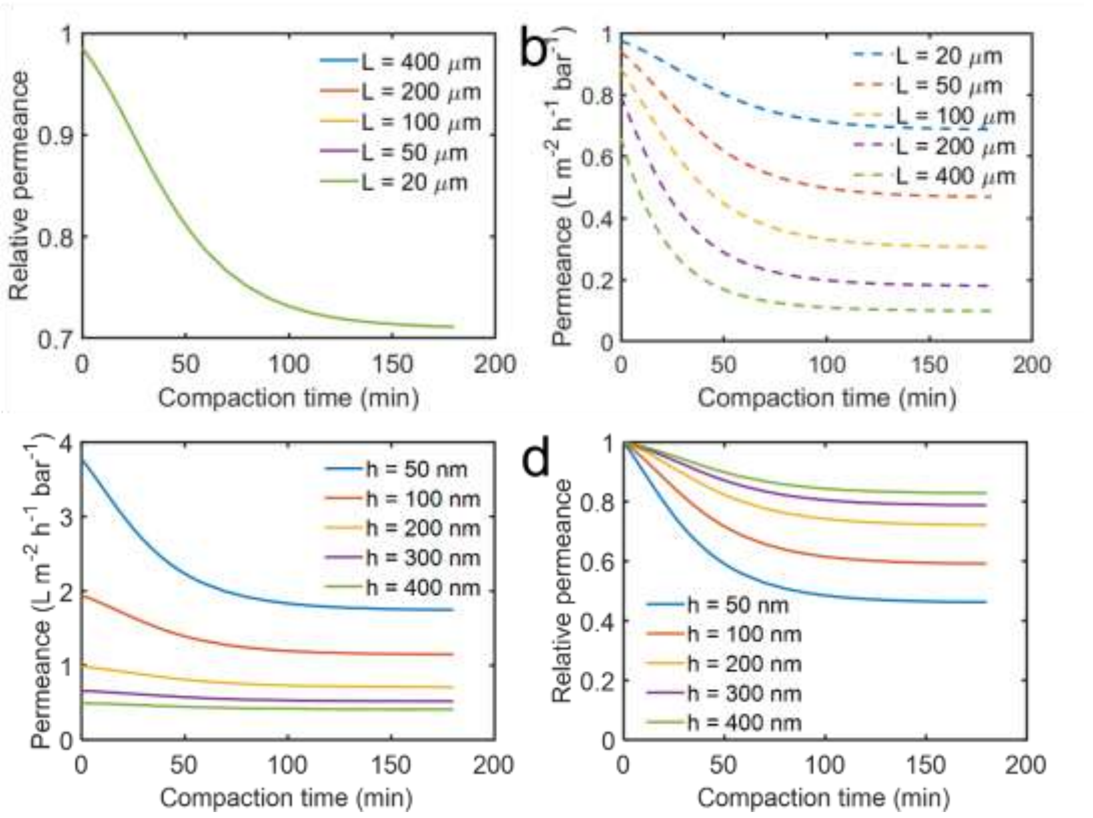

Figure S11. Effect of support thickness and active layer thickness to membrane compaction **(a)** Effect of support thickness (*L*) to membrane compaction for membrane supports with cylindrical pores and foam polymer (C+F, in solid lines). It seems that the support membrane thickness is not affecting the membrane compaction, as the resistance increase in compaction is majorly contributed by the "interface layer" atop the support membrane (**Figure 3e**). The 5 lines overlap with each other. **(b)** Effect of support thickness (*L*) to membrane compaction for supports with foam only structures. (F, in dashed lines). Thicker supports exhibited larger flux decline. **(c)** Effect of active layer thickness on membrane permeance. The baseline value is h = 200 nm and permeance is 1 L m^{-2} h^{-1} bar^{-1}. (**d**) Relative permeance ($P(t)/P(0)$) of the TFC membranes with different active layer thicknesses during compaction. Membranes with thicker active layers experienced less compaction, but their permeance is still lower given the result in **(b)**.

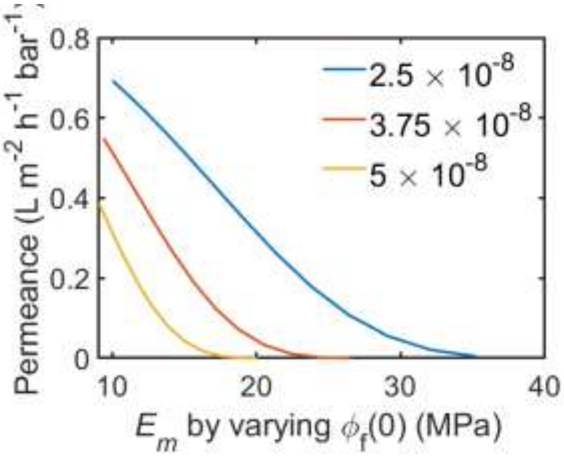

Figure S12. The trade-offs between steady-state modulus and transport properties for TFC membranes with foam-only support polymers. The values in the legends represents the creep compliances in the units of Pa^{-1}.

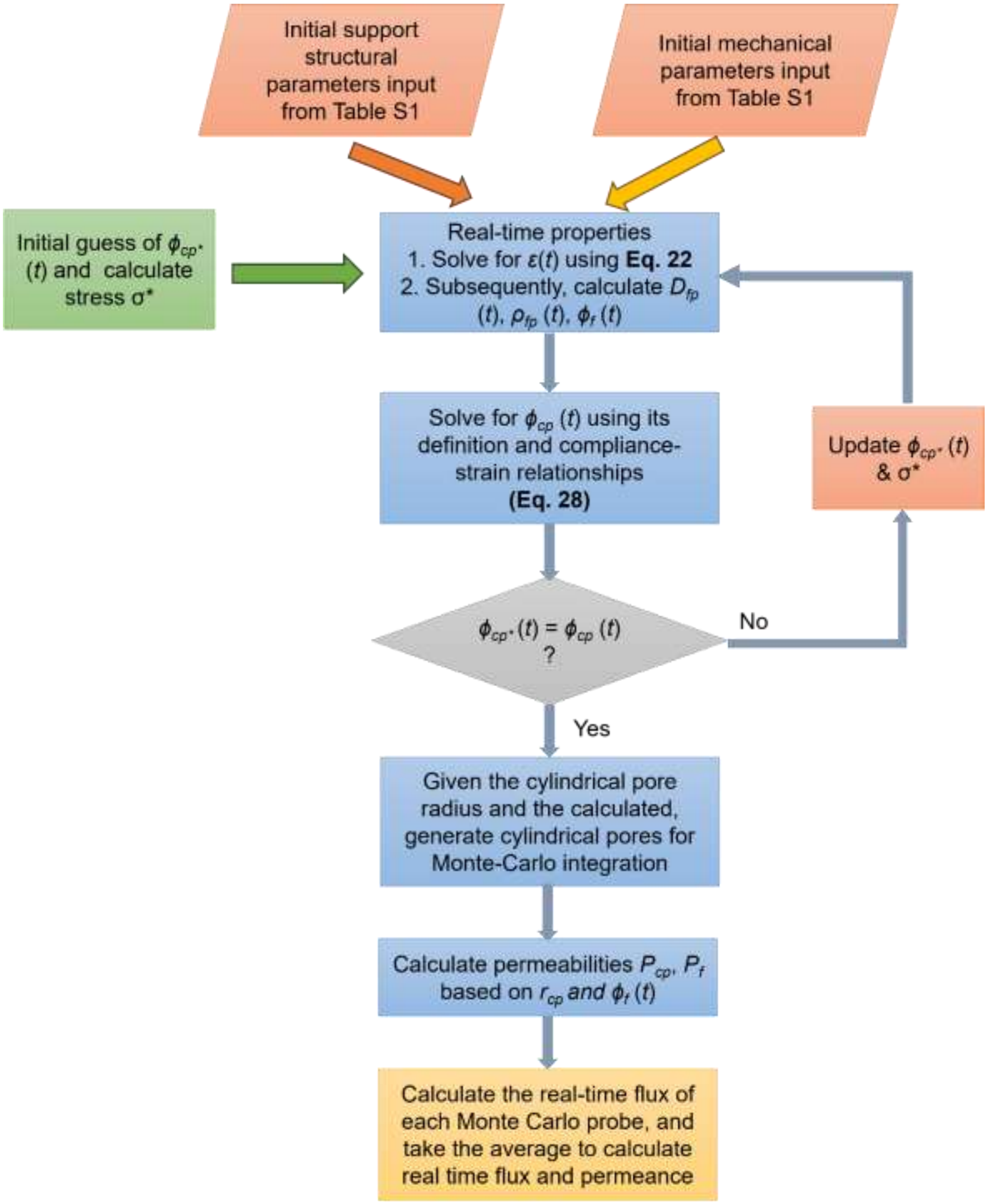

Figure S13. Flowchart for modeling TFC membrane compaction.

Supporting References

1. Pendergast, M. T. M.; Nygaard, J. M.; Ghosh, A. K.; Hoek, E. M., Using nanocomposite materials technology to understand and control reverse osmosis membrane compaction. *Desalination* **2010,** *261* (3), 255-263.

2. Davenport, D. M.; Ritt, C. L.; Verbeke, R.; Dickmann, M.; Egger, W.; Vankelecom, I. F.; Elimelech, M., Thin film composite membrane compaction in high-pressure reverse osmosis. *Journal of Membrane Science* **2020**, 118268.

3. Hussain, Y. A.; Al-Saleh, M. H., A viscoelastic-based model for TFC membranes flux reduction during compaction. *Desalination* **2014,** *344*, 362-370.

4. Zhao, Y.; Lai, G. S.; Chong, J. Y.; Wang, R., Dissecting the structure-compaction-performance relationship of thin-film composite polyamide membranes with different structure features. *Journal of Membrane Science* **2022,** *654*, 120553.

5. Semião, A. J. C.; Habimana, O.; Cao, H.; Heffernan, R.; Safari, A.; Casey, E., The importance of laboratory water quality for studying initial bacterial adhesion during NF filtration processes. *Water Research* **2013,** *47* (8), 2909-2920.

6. Guillen, G. R.; Pan, Y.; Li, M.; Hoek, E. M. V., Preparation and Characterization of Membranes Formed by Nonsolvent Induced Phase Separation: A Review. *Industrial & Engineering Chemistry Research* **2011,** *50* (7), 3798-3817.

7. Dawson, M. A.; Germaine, J. T.; Gibson, L. J., Permeability of open-cell foams under compressive strain. *International Journal of Solids and Structures* **2007,** *44* (16), 5133-5145.

8. Handge, U. A., Analysis of compaction and life- time prediction of porous polymer membranes: influence of morphology, diffusion and creep behaviour. *Polymer International* **2017,** *66* (4), 521-531.

9. Andrews, E.; Gibson, L. J.; Ashby, M., The creep of cellular solids. *Acta materialia* **1999,** *47* (10), 2853-2863.

10. Huang, J.-S.; Gibson, L., Creep of polymer foams. *Journal of materials science* **1991,** *26*, 637-647.

11. Brace, W. F., Permeability from resistivity and pore shape. *Journal of Geophysical Research* **1977,** *82* (23), 3343-3349.

12. Gibson, L. J.; Ashby, M. F., *Cellular solids : structure and properties*. Cellular solids : structure and properties: 1997.

13. Kamal, N.; Ahzi, S.; Kochkodan, V., Polysulfone/halloysite composite membranes with low fouling properties and enhanced compaction resistance. *Applied Clay Science* **2020,** *199*, 105873.

14. Kim, S. H.; Kwak, S.-Y.; Suzuki, T., Positron Annihilation Spectroscopic Evidence to Demonstrate the Flux-Enhancement Mechanism in Morphology-Controlled Thin-Film-Composite (TFC) Membrane. *Environmental Science & Technology* **2005,** *39* (6), 1764-1770.
